# Generation of densely labeled oligonucleotides for the detection of small genomic elements

**DOI:** 10.1016/j.crmeth.2024.100840

**Published:** 2024-08-12

**Authors:** Clemens Steinek, Miguel Guirao-Ortiz, Gabriela Stumberger, Annika J. Tölke, David Hörl, Thomas Carell, Hartmann Harz, Heinrich Leonhardt

**Affiliations:** 1 Faculty of Biology and Center for Molecular Biosystems (BioSysM), Human Biology and BioImaging, Ludwig-Maximilians-Universität München, 81377 Munich, Germany; 2 Department of Chemistry, Ludwig-Maximilians-Universität München, 81377 Munich, Germany

**Keywords:** fluorescence *in situ* hybridization, NOVA-FISH, FISH, STED microscopy, DNA FISH, oligomers, labeling

## Abstract

The genome contains numerous regulatory elements that may undergo complex interactions and contribute to the establishment, maintenance, and change of cellular identity. Three-dimensional genome organization can be explored with fluorescence *in situ* hybridization (FISH) at the single-cell level, but the detection of small genomic loci remains challenging. Here, we provide a rapid and simple protocol for the generation of bright FISH probes suited for the detection of small genomic elements. We systematically optimized probe design and synthesis, screened polymerases for their ability to incorporate dye-labeled nucleotides, and streamlined purification conditions to yield nanoscopy-compatible oligonucleotides with dyes in variable arrays (NOVA probes). With these probes, we detect genomic loci ranging from genome-wide repetitive regions down to non-repetitive loci below the kilobase scale. In conclusion, we introduce a simple workflow to generate densely labeled oligonucleotide pools that facilitate detection and nanoscopic measurements of small genomic elements in single cells.

## Highlights

• Rapid, flexible, and cost-effective synthesis of densely labeled oligonucleotides
• Systematic analysis of distance-dependent dye-dye interactions
• The NOVA workflow selectively yields labeled probe sets from complex probe pools
• NOVA-FISH enables the visualization of sub-kilobase genomic loci

## Motivation

While three-dimensional chromatin conformations can be explored with fluorescence *in situ* hybridization (FISH), the visualization of small genomic loci with high spatial resolution remains challenging. For such applications, programmable oligonucleotides with high brightness are required. To further improve precision and sensitivity, secondary hybridization steps should be omitted. Here, we present a simple, quick, and inexpensive approach to generate labeled FISH probes that carry several fluorophores. Our workflow allows for the free choice of fluorophores, flexible adjustment of labeling density, and selective probe synthesis from large probe pools. With our probes, we reliably detect genomic loci below the kilobase level and examine their topological relationships.

## Graphical abstract

**Figure:**
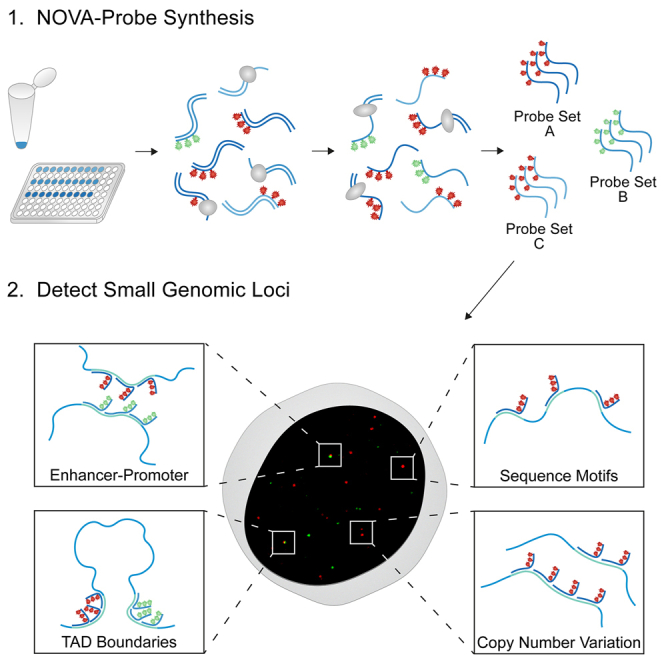

## Teaser

Steinek et al. develop a simple workflow for the enzymatic synthesis of densely labeled oligonucleotides. The described approach allows for free choice of fluorophores and flexible adjustment of labeling density to optimize signal detection. NOVA probes enable the detection of sub-kilobase genomic loci using confocal or super-resolution microscopy.

## Introduction

In recent years, multiple layers of mammalian genome organization ranging from preferential positions of chromosomes in the nucleus to active and inactive compartments and small-scale interactions between individual loci have been uncovered.^1^^,^^2^^,^^3^^,^^4^^,^^5^ An intricate interplay of chromosome territories, topologically associated domains, and regulatory elements defines cellular identity in development and disease.^6^^,^^7^^,^^8^^,^^9^^,^^10^ While current methodologies reliably probe pairwise and multi-contact DNA-DNA interactions, deciphering complex 3D chromatin organization in single cells remains challenging, particularly in the kilobase range.^11^^,^^12^ Thus, there is a growing demand for increased sensitivity to detect and study DNA elements in the 3D context of individual nuclei.

The state of the art for mapping chromatin contacts is chromatin capture assays.^13^^,^^14^^,^^15^^,^^16^^,^^17^ These methods are especially powerful, as they detect contacts within large-scale genomic regions with a resolution ranging from 1 kb down to the nucleosome level, but typically rely on population averages.^18^^,^^19^^,^^20^ However, early efforts to probe chromatin contacts in single cells using chromatin capture assays have revealed extensive cell-to-cell variations within the same population.^21^^,^^22^^,^^23^ Intercell variation of 3D chromatin structures has been observed in multiple imaging studies, which is consistent with the transient nature of chromatin contacts revealed by live-cell imaging.^11^^,^^12^^,^^24^^,^^25^^,^^26^^,^^27^^,^^28^^,^^29^^,^^30^ Therefore, chromatin capture assays need to be complemented with sensitive imaging methods to comprehensively address the dynamics and function of chromatin conformations.

Since their advent, microscopy and fluorescence *in situ* hybridization (FISH) have shed light on the spatial distribution of chromatin in single cells and identified chromosomal abnormalities in malignant cells and tissues.^31^^,^^32^^,^^33^^,^^34^^,^^35^ Although fluorescence microscopy has facilitated studies on large-scale chromatin structures, the detection and resolution of small regulatory elements with traditional FISH methods remains challenging.^24^^,^^36^ In past works, FISH probes have often been generated from bacterial artificial chromosomes (BACs) or yeast artificial chromosomes (YACs) using polymerases in random priming or nick translation reactions.^37^^,^^38^^,^^39^^,^^40^ However, the size of BAC or YAC probes limits the genomic resolution and is, therefore, not suitable for the detection of short regulatory DNA sequences.^41^ Recent advances in synthetic DNA production and the availability of whole-genome datasets have ushered in a new era of oligonucleotide-based FISH (oligoFISH) methodologies.^28^^,^^42^^,^^43^^,^^44^^,^^45^^,^^46^^,^^47^ Variations of oligoFISH utilize barcoded primary pools and fluorescent secondary readout probes to sequentially detect genomic loci. Although this approach has enabled considerable advancements in understanding chromatin architecture, the usage of single-labeled secondary probes limits the detectable target size and spatial resolution. Signal amplification has been achieved through rolling circle amplification,^48^ hybridization chain reaction,^49^^,^^50^ serial ligation of circular DNA (clamp-FISH^51^^,^^52^), branched DNA configurations,^53^ or primer exchange reaction (SABER-FISH^54^). These techniques typically involve multiple hybridization rounds and enable detection of multiple targets, but DNA accessibility and an increased risk of non-specific amplification may complicate the visualization of small genomic elements. We hypothesized that the direct coupling of multiple fluorophores to primary oligonucleotides in combination with the elimination of secondary hybridization steps improves the signal-to-noise ratio at DNA loci of interest.

Here, we introduce a protocol to generate nanoscopy-compatible oligonucleotides with dyes in variable arrays (NOVA probes). Multiple fluorophores are attached to oligonucleotides in a one-step biochemical reaction, thereby considerably shortening the time required for probe generation. The protocol has further been optimized to allow precise control of the labeling density and does not require demanding amplification or purification steps. We applied our probes to detect a variety of genomic loci ranging from large-scale repetitive regions to sub-kilobase single loci using FISH (NOVA-FISH). Compared to previous methods, NOVA-FISH probes can efficiently be produced and allow free choice of fluorophores and flexible adjustment of labeling density to optimize signal detection in super-resolution microscopy.

## Results

### Design and synthesis of NOVA probes

**Figure 1:**
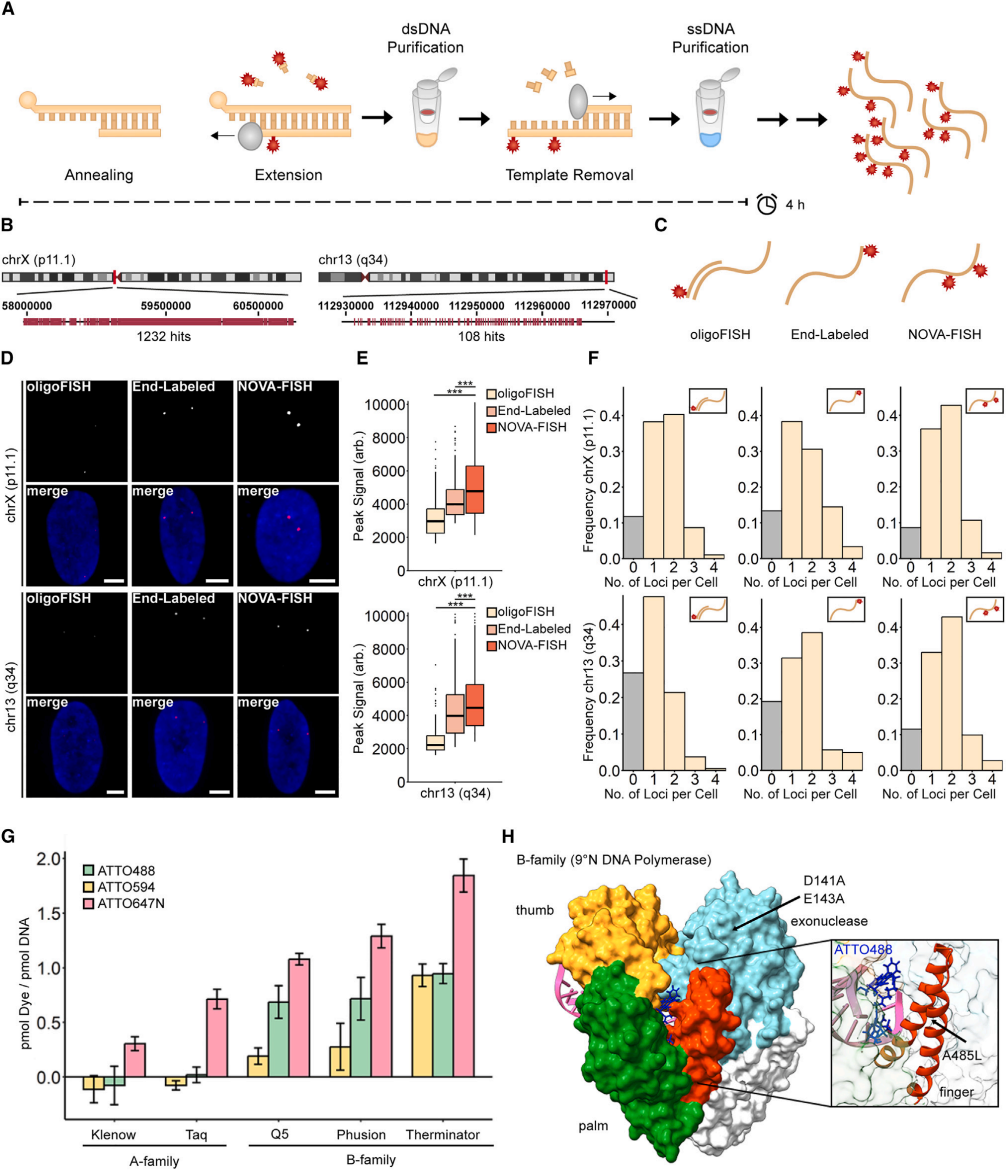
Generating oligonucleotides that carry multiple fluorophores (A) Schematic workflow of the protocol. Primers are annealed to 5′ phosphorylated template strands, and dye-labeled nucleotides are incorporated in a one-step extension reaction. Template strands are then enzymatically removed, and the product is purified. (B) Depiction of the target regions. The target regions contain a unique series of repeats (pink) in chrX (p11.1) or chr13 (q34). Human reference GRCh37/hg19 was used to retrieve coordinates. (C) Comparing three FISH strategies to tag genomic loci. While oligoFISH uses labeled readout strands for detection, end-labeled and NOVA-FISH probes carry fluorophores in their primary sequences. (D) Representative images of both targets detected with oligoFISH, end-labeled probes, or NOVA-FISH. FISH was conducted in IMR-90 cells. Scale bars, 5 μm. (E) NOVA-FISH yields bright FISH signals. Number of detected signals: chrX (p11.1): oligoFISH (*n* = 430), end-labeled (*n* = 420), and NOVA-FISH (*n* = 548); chr13 (q34): oligoFISH (*n* = 292), end-labeled (*n* = 354), and NOVA-FISH (*n* = 413). Datasets were tested for significance using the Wilcoxon rank-sum test with Bonferroni’s correction for multiple testing (∗∗∗*p* < 0.001). (F) NOVA-FISH improves detectability in small genomic loci. Histograms depict the relative number (no.) of chrX (p11.1) or chr13 (q34) foci detected. NOVA-FISH exhibits a significant improvement in the detectability of chr13 (q34) (*p* < 0.001 for NOVA-FISH vs. oligoFISH and NOVA-FISH vs. end labeled, Wilcoxon rank-sum test). Nuclei that have been entirely imaged were included in the analysis. Number of cells analyzed: chrX (p11.1): oligoFISH (*n* = 196), end labeled (*n* = 180), and NOVA-FISH (*n* = 243); chr13 (q34): oligoFISH (*n* = 187), end labeled (*n* = 157), and NOVA-FISH (*n* = 182). (G) Screening substrate preferences of selected DNA polymerases. Polymerases (Klenow exo-, Taq, Q5, Phusion, Therminator) incorporated dCTP-ATTO488, dCTP-ATTO594, or dCTP-ATTO647N into oligonucleotides using a 1:4 molar ratio of dye-labeled to unlabeled nucleotides. Data are represented as mean ± SD. See also Figure S1A. (H) Crystal structure of the 9°N DNA polymerase in complex with DNA and dCTP-ATTO488. The protein is shown in white with highlighted palm (green), thumb (yellow), finger (orange), and exonuclease (cyan) domains. dCTP-ATTO488 was superimposed on the incorporated nucleotide in the complex. The magnified image depicts dCTP-ATTO488 in the binding pocket. See also Figure S2. The figure was generated with UCSF Chimera (v.1.17.3, RRID: SCR_015872) accessing 5OMV.^55^^,^^56^

OligoFISH methods have proven valuable in visualizing genomic regions, but the necessity of multiple hybridization steps and/or the use of expensive, end-labeled probes limit their widespread application in nanoscopy. We reasoned that densely labeled oligonucleotide probe sets could be generated with an enzymatic approach in an efficient and cost-effective manner (Figure 1A). To this goal, we hybridized 5′ phosphate-labeled template strands with short primers followed by primer extension and lambda-exonuclease-mediated template degradation. We synthesized two probes that target a series of repeats on chromosome X (chrX; p11.1) or chr13 (q34) (Figure 1B). Compared with barcoded oligonucleotides and end-labeled probes, our densely labeled oligonucleotides (NOVA probes) significantly improve signal strength (Figures 1C–1E). Moreover, NOVA-FISH exhibits a significant improvement in detectability of the smaller target on chr13 (q34) (*p* < 0.001, Wilcoxon rank-sum test) but not chrX (p11.1). Therefore, NOVA probes are well suited for detecting small genomic loci (Figure 1F).

As our approach depends on the enzymatic incorporation of modified nucleotides into short primers, we compared commonly available DNA polymerases. We measured the incorporation of different dye-labeled nucleotides during extension using commonly available family A (Klenow exo-, Taq) and family B (Q5, Phusion, Therminator) DNA polymerases. Photometric measurements of synthetized probes showed that the highest labeling rates were obtained for all tested modified nucleotides with Therminator DNA polymerase (Figures 1G and S1A–S1C).

Therminator DNA polymerase is a DNA polymerase that has been derived from the euryarchaeon *Thermococcus* sp. 9°N and carries mutations in its exonuclease domain (D141A, E143A) and finger domain (A485L)^57^ (Figure 1H). As a result of these modifications, Therminator DNA polymerase exhibits decreased discrimination for modified nucleotides and has been used to synthesize a variety of unnatural nucleic acids.^58^^,^^59^^,^^60^^,^^61^ To investigate the molecular basis for the observed variations in incorporation efficiencies among our candidates, we modeled dye-labeled nucleotides in different conformations in conjunction with finger domains of family A and B polymerases (Figure S2). We noted possible steric clashes between dye-labeled nucleotides and finger domains of family A members, whereas no such clashes were observed with family B polymerases. Using Therminator DNA polymerase, we determined that probes are robustly generated within an hour (Figure S1D). In addition, our approach allows free choice of fluorophore and flexible adjustment of labeling density (Figure S1E).

FISH probes require a high degree of purity since complementary or unlabeled strands will compete with the labeled probe during hybridization and, thus, reduce signal intensity. To remove unbound primers, free nucleotides, and enzymes, we adapted the buffer conditions to selectively yield double-stranded oligonucleotides after extension (Figures S3A–S3C). Also, unlabeled template DNA might block the synthesized probes and thereby prevent their hybridization with the locus of interest. Therefore, we have introduced phosphate groups at the 5′ ends of template strands to mark them for lambda-exonuclease-mediated degradation (Figure S3D). Using this approach, template DNA was effectively degraded within 30 min (Figure S3E). We then used ethanol-based purification to obtain the single-stranded probe (Figures S3A and S3C). This simple purification strategy yielded all NOVA probes used for microscopic measurements in this work.

After establishing a robust workflow, we assessed the number of incorporated fluorophores in NOVA probes. High-performance liquid chromatography (HPLC) analysis revealed that using a low ratio of modified to unmodified nucleotides (25%) in the synthesis reaction yields distinct elution peaks corresponding to the incorporation of increasing numbers of fluorophores (Figures S3F and S3G).

### Visualizing telomere clustering below the diffraction limit

Next, we sought to utilize the brightness of NOVA probes to visualize telomeres below the diffraction limit. We tagged telomeres with telomere-specific NOVA probes and acquired images using confocal or stimulated emission depletion (STED) microscopy (Figure S1F). We observed clustered telomeres using STED microscopy, which appear as single entities in confocal images. We then applied 3D STED microscopy to gain further insights into the degree of telomere clustering (Figure S1G). Telomeres in the same cells exhibited considerable heterogeneity in their size, and clusters containing multiple telomeres were observed, consistent with previous works.^62^^,^^63^^,^^64^^,^^65^ Next, we analyzed the number of detectable telomeres using confocal or STED microscopy (Figure S1G). In comparison to confocal images, STED microscopy detected, on average, 1.31 times more telomeres (±SD = 0.21), corresponding to clustered telomeres that are only resolved with super-resolution microscopy. Hence, the brightness of NOVA probes supports demanding super-resolution microscopy to visualize nuanced details of genomic loci with high optical resolution.

### Dense labeling does not affect hybridization efficiency but reduces signal strength

**Figure 2:**
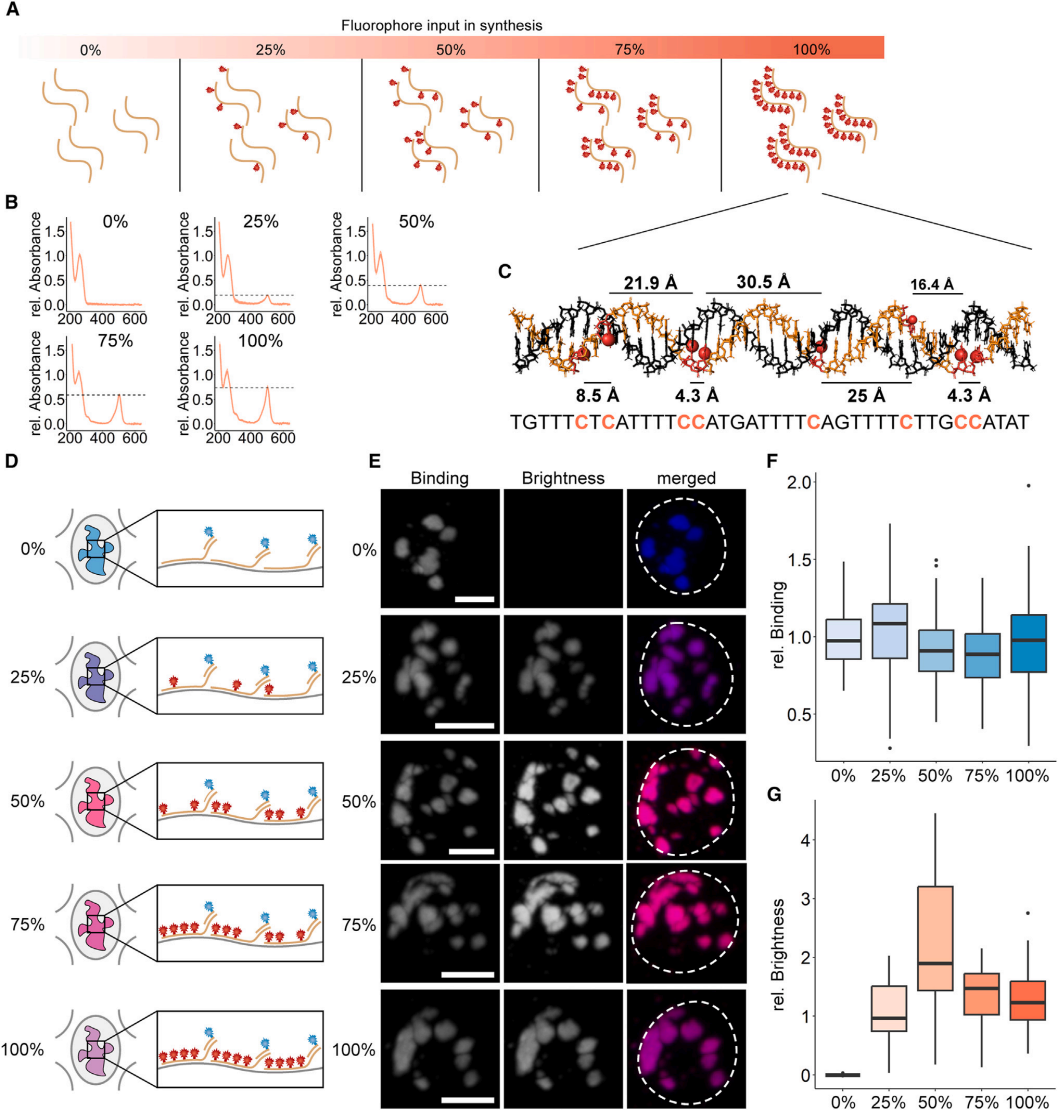
Binding efficiency and brightness of densely labeled probes (A) Modulating labeling densities during NOVA probe synthesis. The labeling density is controlled through the ratio of labeled to unlabeled nucleotide (0%, 25%, 50%, 75%, 100%) in the synthesis reaction. (B) Absorption spectra of probes with increasing labeling density. The absorbance was normalized by the absorption peak at 260 nm. The dotted lines indicate the absorption maximum of the fluorophore. (C) Modeling fluorophore spacing in NOVA-FISH probes bound to major satellites. The B-form duplex formed by a NOVA-FISH probe (beige) and the genomic target (black) is shown. Red nucleotides indicate the locations of modified cytosines, and fluorophores are depicted as red knobs. The normal distance between neighboring fluorophores in the helix is depicted. The figure was created in Pymol v.2.5.5 (RRID: SCR_000305).^66^ (D) Assay to determine the impact of fluorophore number in the probe on hybridization efficiency. NOVA-FISH probes carrying increasing numbers of fluorophores (red) are hybridized with a locus of interest, and dye-labeled secondary strands (blue) are used as a reference. (E) Representative images of major satellites in mouse embryonic stem cells detected with NOVA-FISH probes containing increasing numbers of fluorophores. Scale bars, 5 μm. (F) Binding efficiency is unaffected by dense labeling. The normalized intensity of dye-labeled secondary strands (blue) is depicted. (G) Densely labeled probes exhibit a decrease in fluorescence. Related to (F). The normalized intensity of NOVA-FISH probes (red) is depicted. Number of cells analyzed: 0% (*n* = 149), 25% (*n* = 146), 50% (*n* = 172), 75% (*n* = 147), and 100% (*n* = 165).

As our workflow yields densely labeled probes, we next tested how the presence of multiple dyes in the probe affects hybridization efficiency. To address this, we generated barcoded probes with increasing labeling densities (Figures 2A–2C and S1E). These probes contain dye-labeled sequences that bind to the genome and unlabeled barcodes that hybridize with secondary probes carrying another dye. Using this approach, we can evaluate the brightness of the NOVA probe signal (green) and the relative number of probes localized at the target region (red) (Figures 2D and 2E). We found that increasing the number of dye-labeled nucleotides in the probe did not affect the number of bound probes at the locus of interest, as no notable drop in red signal was observed (Figure 2F). However, the brightness of our probes decreases at high labeling densities (Figure 2G). Consequently, densely labeled probes still bind to the region of interest, but short intermolecular distances between fluorophores impede signal strength (Figure 2C).

**Figure 3:**
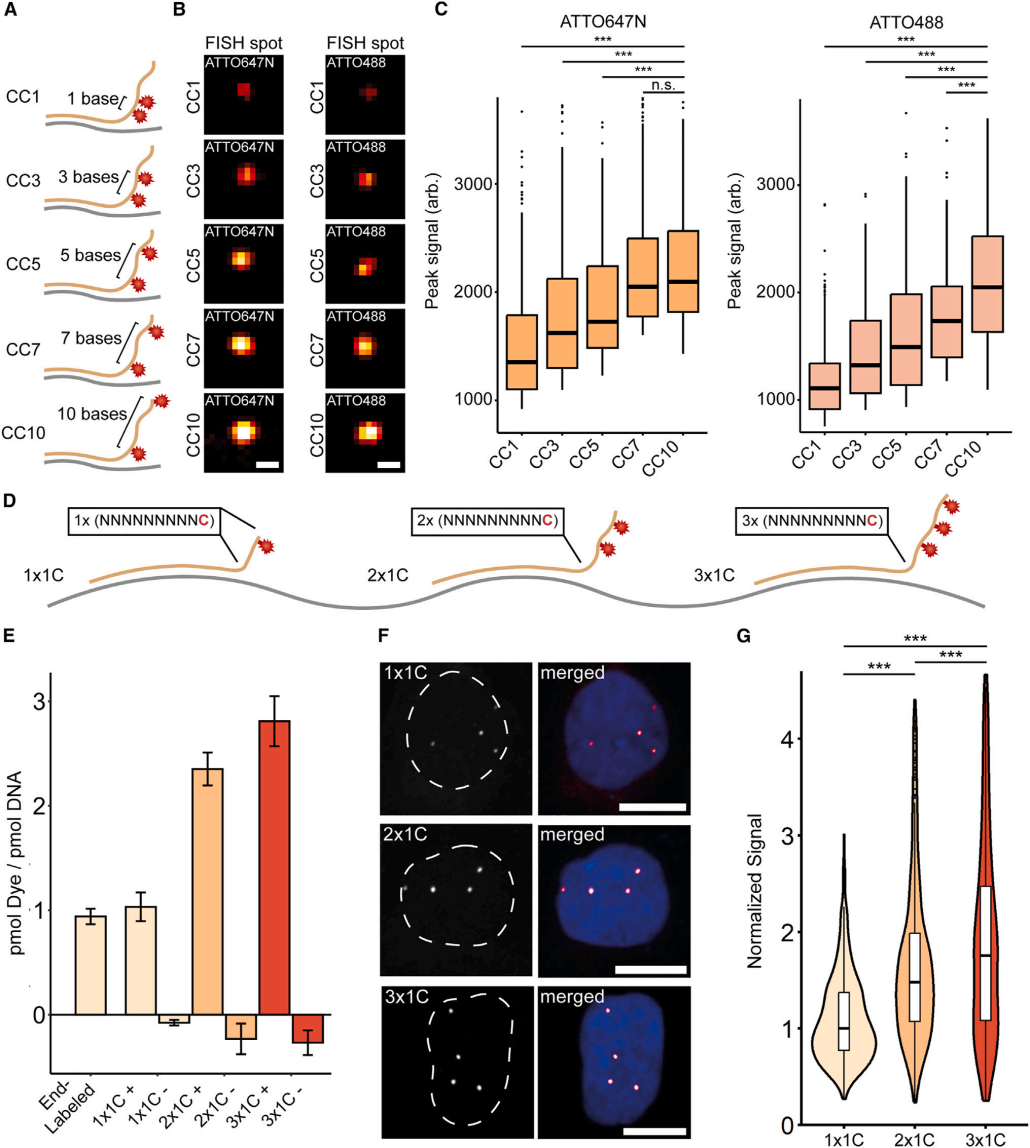
Designing xNOVA probes (A) Design of probes to determine distance-dependent fluorescence quenching. NOVA probes are synthesized to carry two fluorophores with increasing distance in between (1, 3, 5, 7, or 10 bases). (B) Representative images of chr13 (q34) targeted in IMR-90 cells. NOVA probes contain two ATTO488 or ATTO647N molecules. Scale bars, 500 nm. (C) Dye-dye distances impact probe fluorescence. Datasets were tested for significance using the Wilcoxon rank-sum test with Bonferroni’s correction for multiple testing (∗∗∗*p* < 0.001). (D) Design of extended NOVA-FISH (xNOVA) probes. xNOVA probes are extended by labeled 10-mers (NNNNNNNNNC) at their 3′ ends. (E) Synthesizing xNOVA probes with specific fluorophore numbers (1 × 1C, 2 × 1C, 3 × 1C). xNOVA probes were synthesized with a 0% (−ATTO488) or 100% (+ATTO488) ratio of labeled to unlabeled nucleotides. Data are represented as mean ± SD. See also Figure S4A. (F) Representative images of xNOVA probes detecting chr13 (q34) in U2OS cells. Scale bars, 10 μm. (G) Quantification of xNOVA probe signals. Related to (F). The plot depicts the two brightest signals for each cell. Number of foci analyzed: 1C (*n* = 513), 2 × 1C (*n* = 634), and 3 × 1C (*n* = 566). Datasets were tested for significance using the Wilcoxon rank-sum test with Bonferroni’s correction for multiple testing (∗∗∗*p* < 0.001).

We next characterized the impact of dye-dye distances on probe fluorescence. We incorporated two dye molecules into overhangs of probes and increased the distances in between (1, 3, 5, 7, or 10 bases) (Figure 3A). Then, we measured the intensity of probes carrying two ATTO488 or ATTO647N molecules at the FISH spot (Figure 3B). The fluorescence of ATTO488- and ATTO647N-labeled probes increases with greater dye-dye distances (Figure 3C). Therefore, we hypothesize that distance-dependent fluorescence quenching impacts the brightness of densely labeled probes.

### Establishing densely labeled probes with regularly spaced fluorophores

Our previous strategy yields labeled oligonucleotides in an efficient and cost-effective manner but depends on the occurrence of cytosines in the synthesized sequence. Therefore, we modified our workflow to generate extended probes (xNOVA probes) that carry fluorophores in a protruding sequence that does not bind to the genome (Figure 3D).^44^^,^^67^ In this design, fluorophores are regularly spaced in the invariable sequence to avoid distance-dependent fluorescence quenching that might diminish the specific brightness. We synthesized probes that either carried one (1 × 1C), two (2 × 1C), or three (3 × 1C) fluorophores and measured their fluorescence signals at the locus of interest (Figures 3E, 3F, and S4A). The addition of longer sequences (2 × 1C, 3 × 1C) resulted in stronger signals (Figure 3G). With this approach, we observed a steady increase in signal strength at higher labeling densities, arguing against substantial distance-dependent quenching in 3 × 1C sequences (Figures S4B and S4C).

### NOVA-FISH detects non-repetitive genomic loci with kilobase resolution

**Figure 4:**
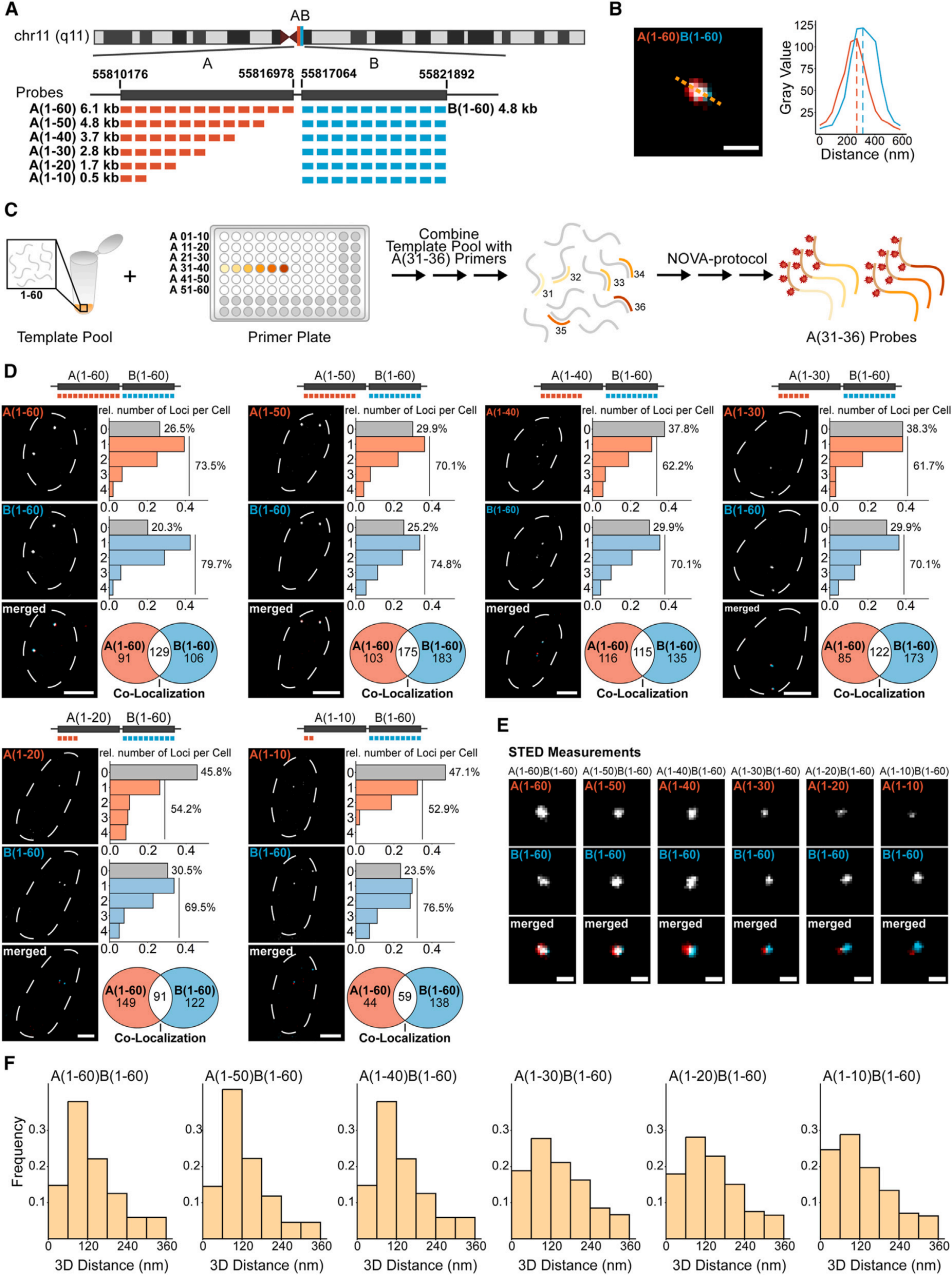
Using xNOVA probes to detect non-repetitive loci at kilobase scale (A) Depiction of the target regions. Two adjacent non-repetitive genomic regions on chr11 (hg19, chr11: 55,810,176–55,816,978, hg19, chr11: 55,817,064–55,821,892) were targeted with 60 xNOVA probes spanning 6.1 or 4.8 kb. In successive experiments, the number of xNOVA probes targeting A was gradually reduced from 60 to 10 probes. 60 xNOVA probes targeting B were used as a reference. (B) Representative STED images of regions “A” and “B” in two colors. A(1–60) was tagged with ATTO594, and B(1–60) harbored ATTO647N. Scale bar, 500 nm. (C) Schematic of selective xNOVA probe synthesis. Probe sets are selectively synthesized through the combination of region-specific primers with common template pools. (D) Detecting kilobase genomic loci using confocal microscopy. 60 probes detecting B were paired with probe sets comprising 60 to 10 individual probes for A. Histograms depict the relative number of detected loci per cell. Venn diagrams depict the number of detected single and co-localizing signals. Scale bars, 5 μm. Number of cells analyzed: A(1–60)B(1–60): *n* = 151; A(1–50)B(1–60): *n* = 153; A(1–40)B(1–60): *n* = 172; A(1–30)B(1–60): *n* = 168; A(1–20)B(1–60): *n* = 124; A(1–10)B(1–60): *n* = 101. (E) Representative z projections of A paired with B in K562 cells. Scale bars, 200 nm. (F) 3D distance of A and B. Number of analyzed spot pairs: 1–60: *n* = 550; 1–50: *n* = 388; 1–40: *n* = 317; 1–30: *n* = 329; 1–20: *n* = 445; 1–10: *n* = 226.

Finally, we tested the limits of NOVA-FISH by detecting small non-repetitive genomic loci with nanoscale precision using STED microscopy (Figure 4A). We designed probe sets to detect non-repetitive neighboring regions on chr11 termed “A” and “B” that have been established in past works.^24^ Probe sets against “A” contained 60, 50, 40, 30, 20, or 10 individual oligonucleotides, while “B” was targeted with 60 probes. The probe sets span 6.1, 4.8, 3.7, 1.7, or 0.5 kb for “A” and 4.8 kb for “B” and yield two adjacent spots (Figure 4B). A characteristic of the NOVA technology is the complete flexibility in probe synthesis, as probes can be selectively amplified from a large pool by adding appropriate primer combinations (Figure 4C). This allows the cost-effective repeated use of one oligonucleotide pool to generate probes against different target regions. Then, we targeted “A” with decreasing numbers of individual probes, maintaining the same set of probes for "B” (Figure 4D). Despite observing a decline in detection frequency with the reduced number of probes detecting "A," we were still able to detect genomic loci as small as 0.5 kb. The ratio of co-localizing spots to total number of spots is in the range of 38%–63% for A and 29%–54% for B. Furthermore, we used STED microscopy to robustly measure distances in all “A” and “B” probe pairs (Figures 4E and 4F). Thus, NOVA-FISH is a reliable tool to detect non-repetitive regions below the kilobase level.

## Discussion

Over the past decade, it became clear that the 3D genome organization contributes to the establishment, maintenance, and change of gene activity.^68^^,^^69^ Chromatin capture assays have identified genome-wide interactions of regulatory elements and have delineated topologically associating domains (TADs).^20^^,^^70^ These findings have traditionally been complemented by FISH-based imaging methods detecting entire genomes and individual chromosomes down to single genomic loci.^28^^,^^47^^,^^71^ However, the optical detection of small genetic elements and the resolution of their spatial relationships at the nanoscale level remains challenging. Here, we developed a simple, rapid, flexible, and cost-effective protocol for the generation of FISH probe sets that are suited for nanoscopic measurements with kilobase resolution. In a recent study, we applied this technique to probe enhancer hijacking events upon tumorigenic translocations.^72^

Small genetic elements are ideally detected with multiple synthetic oligonucleotides that may either be directly labeled or hybridized with secondary, labeled probes. Whereas end-labeled commercial probes are expensive if large and diverse probe pools are used, enzyme-based synthesis is cost effective and flexible but requires the subsequent removal of template strands. While previously, RNA templates were reverse transcribed and subsequently degraded by RNases, we simply removed 5′ phosphorylated DNA templates using lambda exonuclease.^73^ This enzymatic synthesis, including two purification steps, takes under 4 h and yields sets with hundreds of probes for less than 10 €. A detailed cost estimate of oligoFISH, end-labeled, and NOVA probes can be found in Tables S2–S4.

For enzymatic incorporation of dye-labeled nucleotides, we tested commonly available DNA polymerases. We found that B-family DNA polymerases incorporate all used modified nucleotides more effectively than A-family DNA polymerases, such as the Klenow fragment or Taq DNA polymerase. This is consistent with previous structural data of B-family polymerases, attributing their ability to incorporate dye-labeled nucleotides to a larger channel volume, the presence of B-form DNA, and phosphate backbone-mediated protein-DNA interactions.^55^^,^^74^^,^^75^^,^^76^ Among the tested B-family polymerases, Therminator DNA polymerase, having mutations in its exonuclease domain (D141A, E143A) and finger domain (A485L), was best suited for the incorporation of dye-labeled nucleotides.^57^

As even minor FISH projects involve dozens of probes with different dye labels, we used inexpensive, commercially synthesized template pools in combination with plates of bioinformatically optimized, target-specific primers. This approach allows the flexible generation of small to large probe sets coupled with variable dyes. We demonstrate that regions as small as 500 base pairs can be detected and genomic distances of a few kilobases can be measured.

We found that the brightness of probes can be easily adjusted with the ratio of labeled to unlabeled nucleotides in the synthesis reaction. However, the brightness did not linearly increase, due to distance-dependent effects at high labeling densities. To become independent of probe-specific sequences and ensure incorporation of the same numbers of fluorescent nucleotides, we generated extended probes with overhanging, identical sequences (xNOVA). We successfully incorporated fluorophores with a spacing of ten nucleotides but note that distances down to seven nucleotides might be permissible. Our systematic analysis of distance-dependent dye-dye quenching is consistent with a previous study that measured dye-dye interactions in DNA origami.^77^

While current FISH techniques can sequentially label multiple targets, the use of end-labeled probes for secondary hybridization steps reduces signal strength. To enhance signal strength, NOVA probes carrying multiple fluorophores could be employed for secondary hybridization. Moreover, we hypothesize that our workflow is suitable for applications beyond the detection of small genomic loci. Given that oligonucleotides carrying any number of desired fluorophores can be generated, opportunities in the fields of DNA-PAINT, DNA origami, or immunostainings emerge.^78^^,^^79^ In summary, we present a simple, quick, and inexpensive approach to explore the spatial relationships of genetic elements governing the activity of clusters of genes.

### Limitations of study

While NOVA probes enable the detection of sub-kilobase genomic loci, the number of detectable targets is currently limited by the number of distinguishable colors in the microscopy setup. Barcoded probes circumvent this limitation by sequentially binding and releasing labeled readout probes, which, however, leads to a reduction in sensitivity. NOVA probes are not compatible with multiplexed imaging techniques, as they carry fluorophores in their primary sequence. Probing the spatial relationships of a larger number of regulatory elements requires barcoded probes, which could use NOVA probes for readout.

Furthermore, NOVA probes are used for FISH experiments and therefore subject to the same general limitations of hybridization-based methods.^36^^,^^80^ In particular, the same basic trade-offs between the preservation of fine structural details and hybridization penetrance apply.

## STAR★Methods

### Key resources table

**Table:** 

REAGENT or RESOURCE	SOURCE	IDENTIFIER
**Chemicals, peptides, and recombinant proteins**		

5-Propargylamino-dCTP-ATTO-488 (1 mM)	Jena Bioscience	Cat# NU-809-488
5-Propargylamino-dCTP-ATTO-594 (1 mM)	Jena Bioscience	Cat# NU-809-594
5-Propargylamino-dCTP-ATTO-647N (1 mM)	Jena Bioscience	Cat# NU-809-647N-S/L
5-Propargylamino-dCTP-Cy3	Jena Bioscience	Cat# NU-809-CY3-S/L
Diamond™ Nucleic Acid Dye	Promega	Cat# H1181
DiYO-1	AAT Bioquest	Cat# 17580
dNTP Set (100 mM)	Thermo Fisher Scientific	Cat# R0181
Formaldehyde (16%)	Polysciences	Cat# 18814-10
Formamide ≥99.5%	Sigma Aldrich	Cat# F9037
Klenow Fragment (exo-)	Thermo Fisher Scientific	Cat# EP0421
Lambda exonuclease	Thermo Fisher Scientific	Cat# EN0561
Phusion® High-Fidelity DNA Polymerase (Phusion)	New England BioLabs	Cat# M0530 S/L
Q5® High-Fidelity DNA Polymerase (Q5)	New England BioLabs	Cat# M0491 S/L
RNase A	Thermo Fisher Scientific	Cat# EN0531
Taq DNA Polymerase (Taq)	Thermo Fisher Scientific	Cat# EP0401
Therminator™ DNA Polymeras	New England BioLabs	Cat# M0261 S/L

**Critical commercial assays**		

Monarch® PCR & DNA Cleanup Kit	New England BioLabs	Cat# T1030 S/L
NucleoSpin Gel and PCR Clean-up Kit	Macherey-Nagel	Cat# 740609

**Deposited data**		

Uncropped polyacrylamide gels	This paper	https://doi.org/10.17632/nskmtr4h9y.1

**Experimental models: Cell lines**		

IMR-90	Coriell Biorepository	I90-79
J1	ATCC	SCRC-1010
K562	ATCC	CCL-243
U2OS	ATCC	HTB-96

**Oligonucleotides**		

See Table S1		

**Software and algorithms**		

Fiji RRID:SCR_002285	Open Source	https://fiji.sc/
Illustrator CC 2023 RRID:SCR_010279	Adobe	www.adobe.com
ImageJ2 (v.1.54h) RRID:SCR_003070	NIH	www.ImageJ.net/
Microsoft Excel	Microsoft	N/A
Pymol (v.2.5.5) RRID:SCR_000305	Schrödinger	https://pymol.org/2/
UCSF ChimeraX (v.1.17.3) RRID:SCR_015872	UCSF	www.rbvi.ucsf.edu/chimera/

### Resource availability

#### Lead contact

Further information and resource requests can be directed to and will be fulfilled by the lead contact Heinrich Leonhardt (h.leonhardt@lmu.de).

#### Materials availability

NOVA probes were generated using commercially available reagents and services. Sequences and detailed synthesis instructions for generating the probes reported in this study are listed in Table S1 and the Method Details.

#### Data and code availability

• The uncropped polyacrylamide gels have been deposited at Mendeley Data and are publicly available as of the date of publication. An accession number is listed in the Key Resources Table.
• This paper does not report original code.
• Any additional information required to reanalyze the data reported in this paper is available from the lead contact upon request.

### Method details

#### Cell culture

K562 cells were cultivated in Dulbecco’s modified Eagle’s medium (DMEM), 10% fetal bovine serum (FBS), 100 U/mL penicillin and 100 μg/mL streptomycin. U2OS cells were maintained in McCoy’s 5A medium supplemented with 10% FBS, 100 U/ml penicillin, and 100 μg/mL streptomycin at 37 °C in 5% CO_2_. IMR-90 cells were cultured in DMEM, 20% FBS, 1× MEM Non-essential amino acids, and 100 U/mL penicillin, 100 μg/mL streptomycin.

Mouse embryonic stem cells (ESCs) were maintained on culture dishes treated with 0.2% gelatin in DMEM containing 16% FBS, 0.1 mM β-mercaptoethanol, 2 mM L-glutamine, 1× MEM Non-essential amino acids, 100 U/mL penicillin, 100 μg/mL streptomycin, homemade recombinant LIF, and 2i (1 μM PD032591 and 3 μM CHIR99021). For imaging, ESCs were seeded on plates that have been pre-treated with Geltrex diluted 1:100 in DMEM/F12 overnight at 37 °C in 5% CO_2_. Cells were passaged every 2–4 days. All cell lines were regularly tested for Mycoplasma contamination by PCR.

#### Probe design

All generated probe sets are listed in Table S1. NOVA-probes labeling murine major satellites and human repetitive regions (chrX (p11.1) or chr13 (q34), telomeres) were adapted from previously published sequences.^81^^,^^82^ Target regions (“A”: chr11:55810891-55816978 “B”: chr11:55817064-55821430) were chosen in hg38 and 60 unique oligonucleotides were selected and filtered, respectively.^24^ Barcodes of xNOVA-probes containing repetitive sequences (10-mers) were obtained from previous published data.^28^ To generate non-repetitive barcodes, pairs of orthogonal sequences from^83^ were merged. Then, the barcodes were filtered for those containing cytosines every 10 bases and trimmed to the required length.

#### NOVA-FISH Probe synthesis

5′-phosphorylated templates and unlabeled primers were ordered from IDT or Eurofins. Equimolar amounts of 5′-phosphorylated templates and primers (0.10–0.17 nmol each) were combined to a final concentration of 1 μg DNA/μL in 1x ThermoPol Reaction Buffer.

The annealing temperatures were adjusted to the length of the primers. For NOVA-probes (40 nt long templates, 20 nt long primers), the sample was heated up to 95°C for 5 min followed by a stepwise cool-down (1°C/minute) to room temperature. For xNOVA-probes or xNOVA-pools (50–70 nt long templates, 40 nt long primers) the sample was heated up to 95°C for 5 min followed by a stepwise cool-down (1°C/2 min) to 60°C. Complex xNOVA-probe sets were synthesized by adding 2-fold excess of primer sets (e.g., primer 31–40 against “A”) to the template pool.

NOVA- and xNOVA-probes were synthesized by adding 2–4 μg annealed DNA (2–4 μL of the solution) to a reaction mixture containing 0.25 mM dATP/dGTP/dTTP each, 0–0.25 mM dCTP, 0–0.25 mM dye-labeled dCTP and 3 U Therminator DNA polymerase in 1x ThermoPol Reaction Buffer (10 μL total volume). The ratios of dye-labeled dCTP to unlabeled dCTP varied depending on the desired labeling density. The reaction was carried out for 60 min at 72°C.

To remove single-stranded DNA, NucleoSpin Gel and PCR Clean-up Kit (Macherey-Nagel) was used according to the manufacturer’s instructions. 9 volumes of buffer NTI (provided by the manufacturer) were added to one volume of sample before binding. After washing, the DNA was eluted twice in 22 μL ddH_2_O (44 μL final volume). In the next step, 5′-phosphorylated strands were removed by adding 1 μL Lambda exonuclease (10 U/μL) and 5 μL Lambda exonuclease reaction buffer (10x) to a final volume of 50 μL and incubating for 30 min at 37°C. The synthesized probes were then purified using the Monarch PCR & DNA Cleanup Kit (New England BioLabs) according to the manufacturer’s instructions and the quality was verified on denaturing 12–16% polyacrylamide gels.

#### Quality control and purification

The absorbance of samples was measured at 260 nm and 488 nm, 596 nm, or 647 nm depending on the incorporated fluorophore using a Nanodrop 2000 spectrophotometer (Thermo Fisher Scientific). To assess the quality of generated probes, samples were denatured in 90% formamide, 0.5% EDTA, 0.1% Xylene cyanol, 0.1% bromophenol blue and loaded onto a 12% polyacrylamide gel containing 6 M urea. The gel was incubated in 1x TBE buffer containing 1x Diamond Nucleic Acid Dye for 30 min at room temperature to visualize single-stranded DNA.

Complex probe sets labeling target region “A” or “B” were further purified following the “crush and soak” method with adaptations.^84^ Briefly, segments of the polyacrylamide gel containing the band of interest were cut out and 2 volumes of a buffer containing 10 mM magnesium acetate tetrahydrate, 0.5 M ammonium acetate, 1 mM EDTA (pH 8.0) and 0.1% (w/v) SDS was added followed by incubation at 37°C for 16–24 h. The samples were then centrifuged at 13000 x g for 1 min and the supernatant was once more purified using the Monarch PCR & DNA Cleanup Kit (New England BioLabs). We expect the “crush and soak” method to improve signal strength if low labeling densities are used during extension.

#### Polymerase Screens

Polymerases were tested for their ability to incorporate dCTP-ATTO488, dCTP-ATTO594, or dCTP-ATTO647N into oligonucleotides. The maximum number of incorporated dCTP-dye in the probe was eight (CATCCTGAAGGAATGGTCCATG**C**TTA**CC**TGGG**CCC**AT**CC**T).

For detailed information about the reaction conditions see Table S2. 0.1 nmol annealed DNA was added to the recommended reaction mixtures (10 μL final volume) and 5 U of the respective polymerase was added. The following temperatures were used during synthesis: Klenow exo-at 30°C, Taq at 64°C, Q5 at 64°C, Phusion at 64°C, Therminator DNA polymerase at 72°C. All reactions were carried out for 60 min and the reactions were stopped by adding 1 μL 0.5 M EDTA. We did not observe notable differences in incorporation efficiency between the reported results and reactions carried out at higher temperatures (Klenow exo-at 37°C, Taq at 72°C, Q5 at 72°C, Phusion at 72°C, Therminator DNA Polymerase at 75°C) (figure not shown). The absorbance of synthesized products was measured at 200–700 nm on Nanoquant plates using a Tecan Spark microplate reader (Tecan) and choosing the following dye-correction factors: CF_260_(ATTO488): 0.22, CF_260_(ATTO594): 0.22, CF_260_(ATTO647N): 0.04. The depicted data contained at least two measurements per biological replicate.

#### HPLC

HPLC was used to characterize the number of incorporated fluorophores in NOVA probes with low fluorophore input in synthesis (Figures S3G and S3H). ATTO594-labeled and ATTO647N-labeled probes (0.31 nmol or 0.34 nmol) were analyzed and purified by reverse-phase HPLC using an Agilent Technologies 1260 Infinity II System with a G7165A detector equipped with an EC 250/4 Nucleodur 100-3 C18ec column from Macherey Nagel. A gradient of 0–80% of buffer B in 45 min at 60°C with a flow rate of 1 mL/min was applied. The following buffer system was used: buffer A: 100 mM NEt_3_/HOAc, pH 7.0 in H_2_O and buffer B: 100 mM NEt_3_/HOAc, pH 7.0 in H_2_O/MeCN 20/80. The fractions of each signal peak were combined, and the solvents were concentrated by vacuum centrifugation.

#### Sample preparation and fluorescence *in situ* hybridization

Fluorescence *in situ* hybridization was performed as previously described.^24^ Adherent cells were grown overnight on glass coverslips (1.5, 18 × 18 mm, Marienfeld), washed twice with 1x Dulbecco’s Phosphate Buffered Saline (PBS), and fixed using osmotically balanced and methanol-free 4% formaldehyde for 10 min at room temperature. Alternatively, PBS-washed suspension cells were resuspended in a small volume of PBS at a density of 1 million cells per mL and applied to poly-L-lysine coated glass coverslips followed by the addition of methanol-free 4% formaldehyde for 10 min at room temperature. The slides were washed twice in 1x PBS for 5 min and the cells were permeabilized in 1X PBS containing 0.5% Triton X-100 for 15 min. After two successive washing steps in 1x PBS, 0.1 M HCl was added to the slides for 5 min. The slides were washed twice with 2 x SSC and were placed onto a solution containing 1 μg/mL RNase for 30 min at 37°C in a wet chamber. Then, adherent or suspension cells were pre-equilibrated in 2x SSC containing 50% formamide for 60 min or overnight, respectively, inverted onto 8 μL of hybridization solution, and sealed with rubber cement (Marabu). The slides were placed on a heat block set to 81°C for 3 min and incubated at 37°C overnight (16–20 h).

On the second day, slides were washed twice with 2x SSC for 15 min followed by two successive 7-min washes in 0.2x SSC containing 0.2% Tween 20 at 56°C. Then, slides were washed with 4x SSC containing 0.2% Tween 20 and with 2x SSC for 5 min, respectively.

For oligoFISH probes, a second hybridization step was performed for 30 min at room temperature. The slides were then washed once with 2x SSC containing 30% formamide for 7 min at 37°C, twice with 2 x SSC for 5 min, once with 0.2X SSC containing 0.2% Tween 20 at 56°C, once with 4x SSC containing 0.2% Tween 20 for 7 min at room temperature and once with 2x SSC for 5 min.

DNA was counterstained with DAPI (1 μg/mL in 2x SSC) for 10 min and washed twice with 2x SSC. For STED microscopy, nuclei were counterstained with or DiYO-1 (12.5 nM in 2x SSC) for 30 min and washed twice with 2x SSC for 5 min, respectively. Coverslips were mounted on microscopic slides with MOWIOL (2.5% DABCO, pH 7.0), dried for 30 min, and sealed with nail polish.

#### Image acquisition

Confocal images were acquired using a Nikon TiE microscope equipped with a Yokogawa CSU-W1 spinning-disk confocal unit (50 μm pinhole size), an Andor Borealis illumination unit, Andor ALC600 laser beam combiner (405 nm/488 nm/561 nm/640 nm), Andor IXON 888 Ultra EMCCD camera, and a Nikon 100×/1.45 NA oil immersion objective. The microscope was controlled by software from Nikon (NIS Elements, ver. 5.02.00).

Super-resolution was carried out on a 2C STED 775 QUAD Scan microscope (Abberior Instruments) equipped with a 100x 1.4 NA UPlanSApo oil immersion objective lens (Olympus), 3 pulsed excitation lasers (485 nm, 594 nm, 640 nm) and a pulsed depletion laser of 775 nm.

#### 3D STED microscopy of telomers using adaptive illumination

To avoid photobleaching NOVA-FISH stained telomers of IMR90 cells in 3D, stacks were acquired using adaptive illumination STED microscopy.^85^ Cells were recorded using a pixel size of 30 nm, z-steps of 80 nm, a 10 μs dwell time, and a pinhole size of 50 μm.

#### Automated STED microscopy for two-color NOVA-FISH

Automated STED microscopy was performed according to Brandstetter et al..^24^ The acquisition of 3D confocal stacks was automated using home-written Python scripts to control the microscope. Spots within confocal scans were detected using a Laplacian-of-Gaussian blob detector for both channels. Detected spots no further apart than 5 pixels from another spot in the other channel were imaged using 3D STED settings. This process was repeated for each detected spot pair within a confocal scan. Following a spiral pattern, the stage was moved to the next overview to repeat the confocal scan and the subsequent detailed STED acquisition until a specified amount of time elapsed.

#### Image analysis

For the analysis of the effects of labeling density (Figures 2E–2G), cells in confocal z-stacks of major satellites were segmented first via automatic thresholding in a z-maximum projection of the DAPI channel followed by a second round of thresholding in the 640nm (rel. binding) channel to segment major satellites. In the segmented areas, intensities of both the 488nm (rel. brightness) and the 640nm (rel. binding) channels were measured, background, determined by a manually selected ROI outside the cells, was subtracted, and measurements were averaged (median) per cell. For the plots, measurements were normalized to the intensity at 100% for the binding channel and at 25% for the brightness channel. Analysis was carried out using Fiji.^86^

For analysis of image data of repetitive and non-repetitive loci (in Figures 1D–1F; Figures 3B and 3C, Figures 3F and 3G; Figures 4D; S4C), nuclear segmentation maps of confocal images stained with DAPI or DiYO-1 were obtained using Otsu thresholding. FISH spots within segmentation maps were detected using a Laplacian-of-Gaussian blob detector (Figure 3F and 3G; Figure S4C). Alternatively, FISH spots were detected in each channel using RSFISH^87^ and detection threshold parameters were adjusted if necessary (in Figures 1D–1F; Figures 3B and 3C; Figure 4D). Segmentation maps were used to calculate the total number of spots per cell, to obtain the mean background signal within single nuclei to calculate the spot signal over the nucleus background, and the signal-to-noise ratio of single spots. For Figure 4D, distances <500 nm between A and B were considered co-localizing.

Analysis of automated STED measurements of FISH spot pairs was performed as previously described.^24^ Automated image acquisition generated large quantities of data requiring an additional quality control step. To filter out low-quality images, we used a machine learning-based classifier (Random Forest) to label images as “good” or “bad”. The classifier was trained with a ground truth dataset created by an experienced scientist who manually sorted images.

Detailed spot analysis was performed on images passing this QC step. Subpixel localization of FISH spots in both channels was performed by fitting a multidimensional Gaussian function plus a constant background using the Levenberg-Marquardt algorithm. The peak height of the fitted Gaussians was used to determine spot intensity.

### Quantification and statistical analysis

The experiments shown in this study were performed as three biologically independent experiments (*n* = 3) and the figures contain pooled data. No statistical methods were used to predetermine the sample size. Images depicted are representative images from the experiments and dotted lines indicate the outlines of the cells. Data plotted as boxplots indicate the 25th and 75th percentiles, with the whiskers showing the minima and maxima (5th and 95th percentiles), black circles indicating the outliers, and the horizontal line showing the median. Some data are plotted in bar graphs as the mean ± SD. Data was normalized by the median of the first depicted condition in the replicates, if not stated otherwise. Significance levels were tested by non-parametric two-sided Wilcoxon tests or pairwise comparisons using the Wilcoxon rank-sum test with Bonferroni’s correction for multiple testing (∗ = *p* < 0.05, ∗∗ = *p* < 0.01, ∗∗∗ = *p* < 0.001). Sample sizes for all of the graphs are indicated in the figures or figure legends.

## Supplemental information

Document S1. Figures S1–S4 and Tables S2–S4

Table S1. DNA sequences used for NOVA probe synthesis, barcoded oligonucleotides, and end-labeled probes, related to STAR Methods

Document S2. Article plus supplemental information
