# Supplementary material for: Generation of densely labeled oligonucleotides for the detection of small genomic elements
Source: Cell Rep Methods. 2024 Aug 12;4(8):100840. doi: 10.1016/j.crmeth.2024.100840 (PMC11384094; doi:10.1016/j.crmeth.2024.100840)
Supplement: Document S1. Figures S1–S4 and Tables S2–S4 [file mmc1.pdf]

Cell Reports Methods, Volume *4*

Supplemental information

Generation of densely labeled oligonucleotides for the detection of small genomic elements

Clemens Steinek, Miguel Guirao-Ortiz, Gabriela Stumberger, Annika J. Tölke, David Hörl, Thomas Carell, Hartmann Harz, and Heinrich Leonhardt

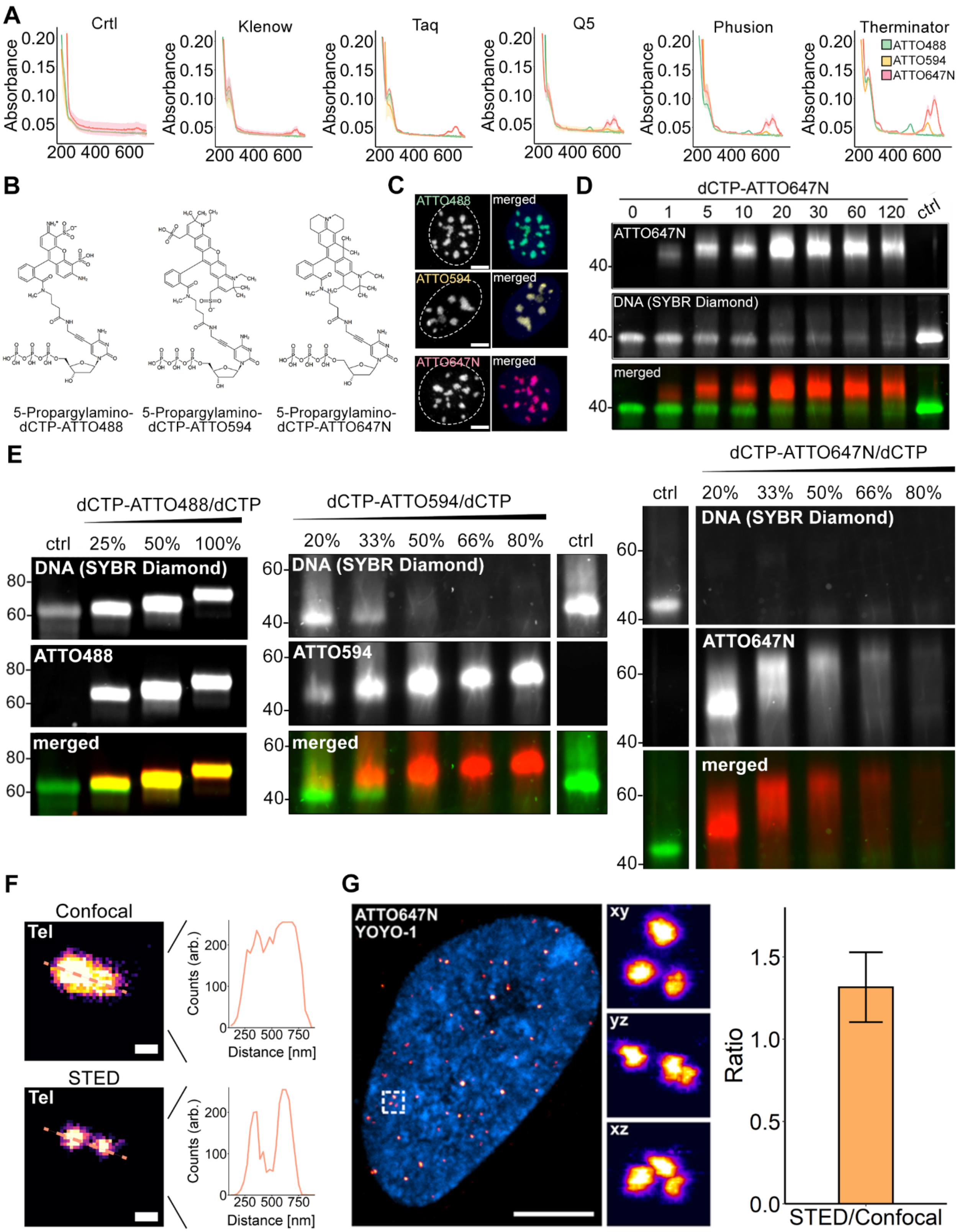

A
Crtl
Klenow
Taq
Q5
Phusion
Therminator
Absorbance
0.20
0.15
0.10
0.05
200 400 600
ATTO488
ATTO594
ATTO647N
B
5-Propargylamino-dCTP-ATTO488
5-Propargylamino-dCTP-ATTO594
5-Propargylamino-dCTP-ATTO647N
C
ATTO488
ATTO594
ATTO647N
merged
D
dCTP-ATTO647N
0 1 5 10 20 30 60 120 ctrl
ATTO647N
DNA (SYBR Diamond)
merged
40-
E
dCTP-ATTO488/dCTP
ctrl 25% 50% 100%
dCTP-ATTO594/dCTP
20% 33% 50% 66% 80% ctrl
dCTP-ATTO647N/dCTP
ctrl 20% 33% 50% 66% 80%
DNA (SYBR Diamond)
ATTO488
ATTO594
ATTO647N
merged
80-
60-
40-
F
Confocal
STED
Tel
Counts (arb.)
200
100
0
250 500 750
Distance [nm]
G
ATTO647N
YOYO-1
xy
yz
xz
Ratio
1.5
1.0
0.5
0.0
STED/Confocal

Supplementary Figure 1 Synthesizing NOVA-probes harboring different fluorophores. Related to Figure 1. (A) Absorption spectra of synthesized probes. The line depicts the mean absorption and the area indicates the standard deviation of three experiments with at least two separate measurements (n = 3). The incorporated nucleotides are indicated as follows: ATTO488 = green, ATTO594 = yellow, ATTO647N = red. (B) Structural formulas of used modified nucleotides. Dyes (ATTO488, ATTO594, ATTO647N) are linked to 5C positions of cytosines. The structures were provided by the manufacturer (Jena Bioscience). (C) Labeling major satellites in J1 cells with NOVA-FISH using three different fluorophores. All probes (maj. Sat.-ATTO488, maj. Sat.-ATTO594, maj. Sat.-ATTO647N) were generated using a one to four molar ratio of dye-labeled to unlabeled nucleotides. Scale bars, 5 µm (D) Therminator DNA polymerase effectively generates dye-labeled probes. The synthesis was carried out between 0-120 minutes using 0.15 nmol DNA and 3 U Therminator polymerase. Fluorescent DNA (ATO647N) and DNA (SYBR-Diamond) are shown in red and green, respectively. (E) Generating probes with different labeling densities. Left: Synthesis of ATTO488-labeled probes detecting major satellites. Middle: Synthesis of ATTO594-labeled probes targeting a subtelomeric region in chromosome 13. Right: Synthesis of ATTO647N-labeled probes detecting a subtelomeric region in chromosome 13. Different dye-labeled dCTP to dCTP ratios were used (25%, 50%, 100% or 20%, 33%, 50%, 66% 80%). Unlabeled probes were used as a control. Fluorophores and stained DNA (SYBR Diamond) are shown in red and green, respectively. (F) Super-resolution microscopy uncovers clustered telomeres. Representative image of two clustered telomeres using confocal microscopy or STED microscopy. Scale bars, 200 nm. (G) Telomere clustering is a common phenomenon in mitotic cells. Representative image of telomeres in IMR-90 cells using 3D-STED microscopy. Detailed view (white box) in three dimensions. Scale bar, 5 µm. Ratio of telomeres detected with STED or confocal microscopy in the same cells is depicted. Telomeres were counted in 10 individual cells from three experiments (n = 3). The black lines depict the mean.

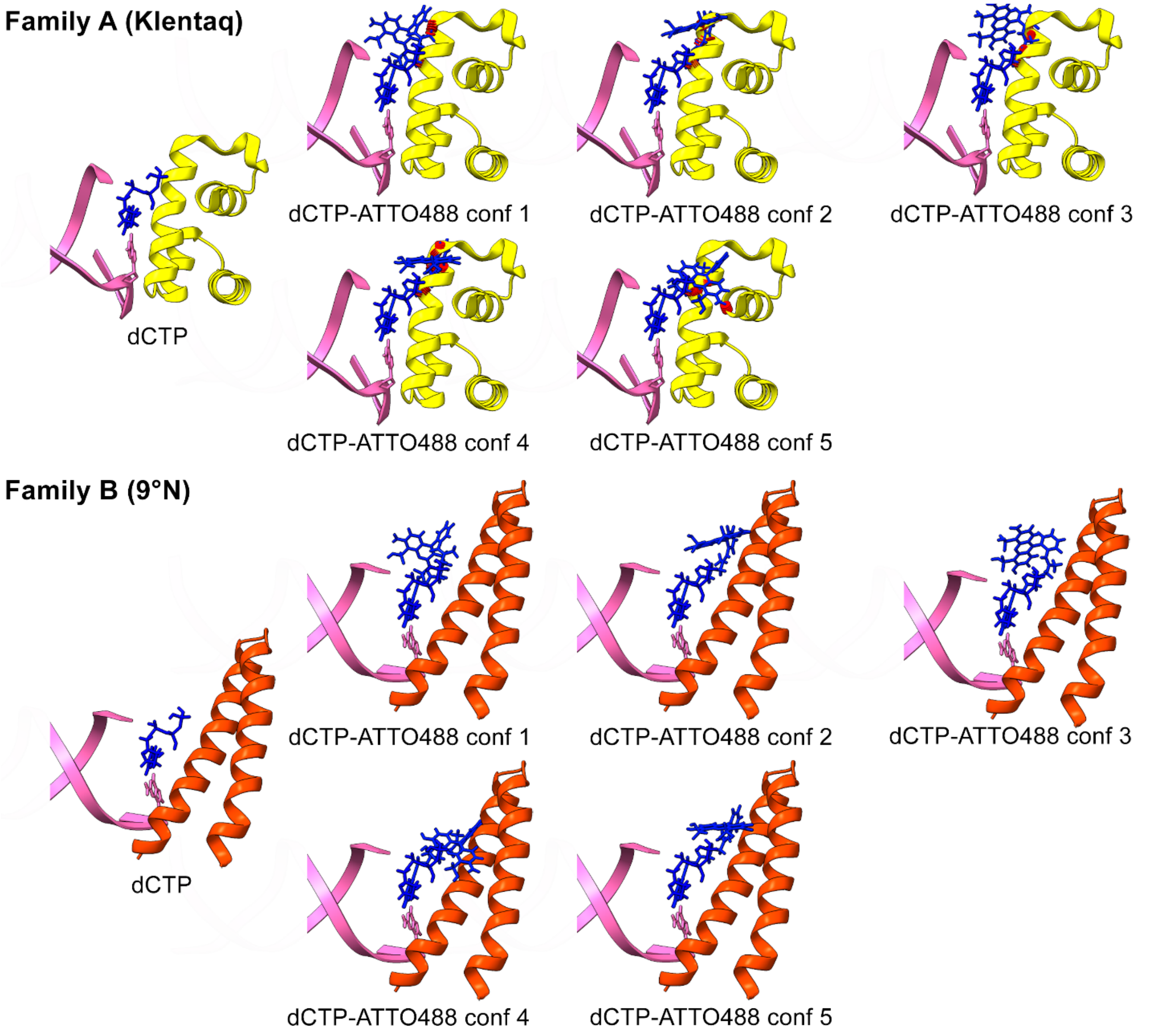

Supplementary Figure 2 Modeling the proximity of dCTP-ATTO488 to family A or family B finger domains. Related to Figure 1. Finger domains are shown in yellow (Klentaq) and orange (9°N DNA polymerase) with DNA in pink and the substrate in blue. Five different conformations of dCTP-ATTO488 (conf1-5) were superimposed on cytosine. Distances between the finger domain and dCTP-ATTO488 < 1 Å are depicted as red knobs. The finger domains are shown in the closed state. The figure was generated with UCSF Chimera (v.1.17.3, RRID:SCR_015872) by using the structures 3RTV and 5OMV [S1-S3].

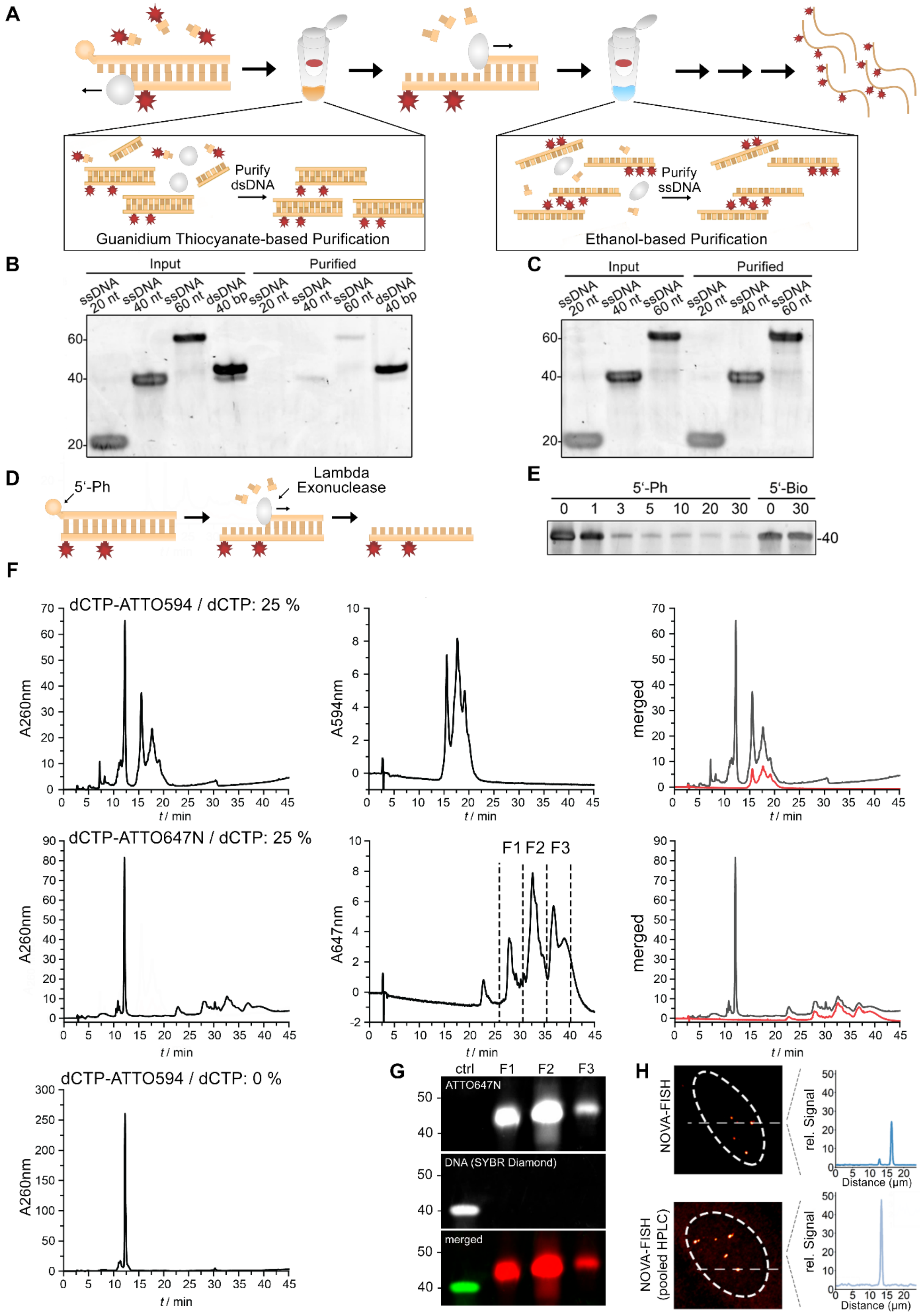

A
Purify dsDNA
Guanidium Thiocyanate-based Purification
Purify ssDNA
Ethanol-based Purification
B
Input
Purified
ssDNA 20 nt
ssDNA 40 nt
ssDNA 60 nt
dsDNA 40 bp
C
D
5'-Ph
Lambda Exonuclease
E
5'-Ph
5'-Bio
0 1 3 5 10 20 30 0 30
-40
F
dCTP-ATTO594 / dCTP: 25 %
A260nm
A594nm
merged
t / min
dCTP-ATTO647N / dCTP: 25 %
A647nm
F1 F2 F3
dCTP-ATTO594 / dCTP: 0 %
G
ctrl F1 F2 F3
ATTO647N
DNA (SYBR Diamond)
merged
H
NOVA-FISH
NOVA-FISH (pooled HPLC)
rel. Signal
Distance (µm)

Supplementary Figure 3 Optimization of probe purification. Related to STAR Methods. (A) Schematic highlighting two purification steps in the protocol. While guanidium thiocyanate-based purification enriches double-stranded DNA, ethanol-based purification purifies single-stranded oligonucleotides. (B) Guanidium thiocyanate-based purification yields double-stranded DNA. ssDNA (20 nt, 40 nt, 60 nt) and dsDNA (40 bp) were loaded before (input) and after (purified) purification. (C) Ethanol-based purification yields single-stranded DNA. ssDNA (20 nt, 40 nt, 60 nt) was loaded before (input) and after (purified) purification. (D) Schematic of lambda exonuclease-mediated degradation. Lambda exonuclease selectively removes 5'-phosphorylated (5'-Ph) strands. (E) Removal of the 5'-phosphoryated template. Templates were incubated with 10 U lambda exonuclease. 5-biotinylated oligonucleotides were used as a control. (F) HPLC of NOVA-FISH probes. Probes were synthesized with Therminator DNA Polymerase using a one to four molar ratio of dye-labeled to unlabeled nucleotides (highest possible number of incorporated fluorophores: 8). The absorptions of ATTO594-labeled and ATTO647N-labeled probes were measured at 260 nm / 594 nm and 260 nm / 647 nm, respectively. Local peaks indicate probes with varying numbers of fluorophores. Probes synthesized without modified nucleotides were used as a control (left). (G) Polyacrylamide gel reveals a visible shift between populations with different fluorophore numbers. Three fractions (F1, F2, F3) were isolated from (F) through preparative HPLC. The precise mechanism of SYBR-Diamond-ssDNA interactions has yet to be elucidated but we observed that the presence of fluorophores in the probe impacts staining efficiency. Unlabeled DNA was used as a control (ctrl). (H) Side-by-side comparison of unpurified and purified NOVA-probes. To evaluate the benefits of HPLC purification, we conducted FISH in U2OS cells using NOVA-probes before and after HPLC purification. The relative signal intensity along the dotted line is depicted. Images were acquired at the same conditions.

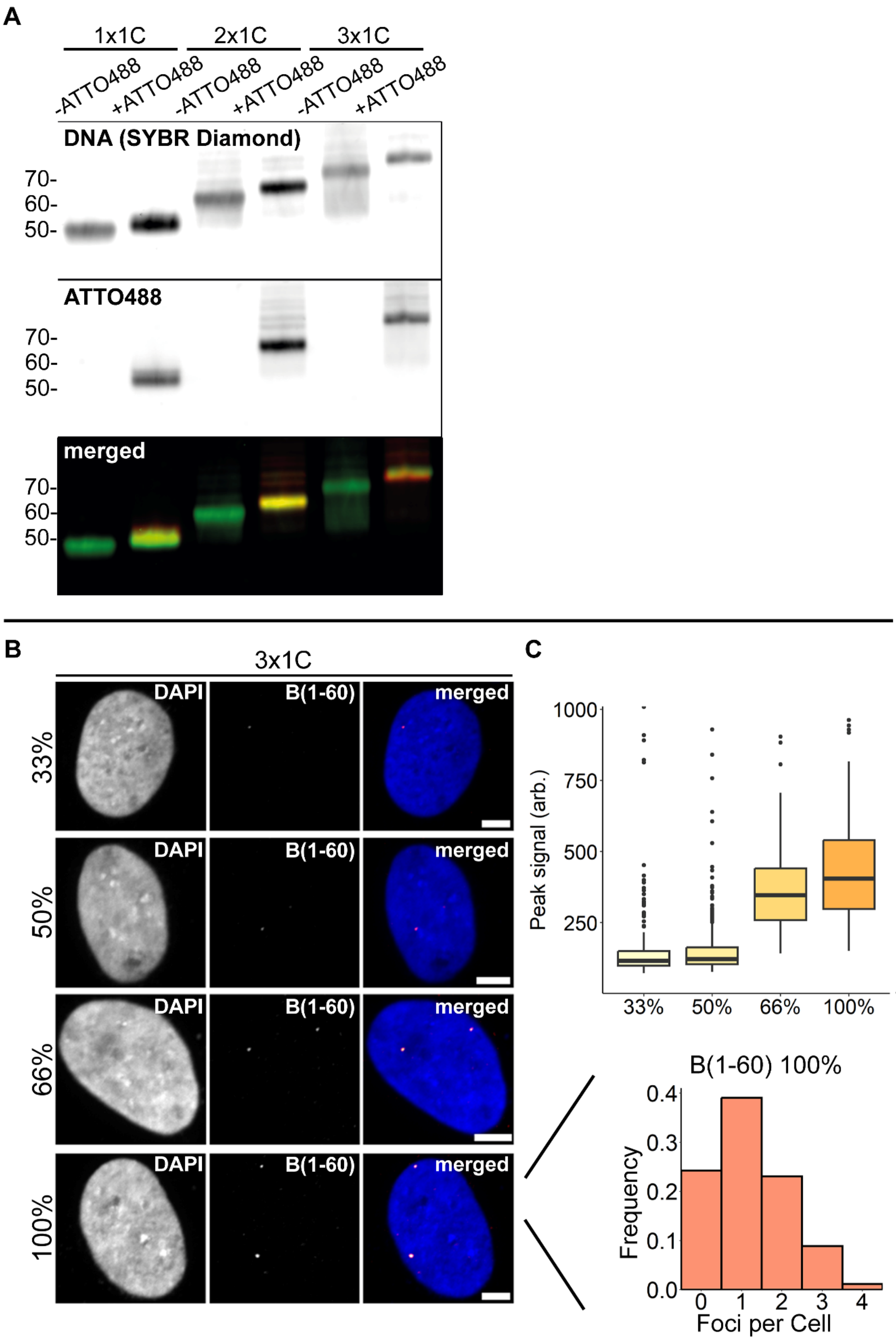
A
1x1C
2x1C
3x1C
-ATTO488
+ATTO488
-ATTO488
+ATTO488
-ATTO488
+ATTO488
DNA (SYBR Diamond)
70-
60-
50-
ATTO488
70-
60-
50-
merged
70-
60-
50-
B
3x1C
DAPI
B(1-60)
merged
33%
50%
66%
100%
C
Peak signal (arb.)
1000
750
500
250
33%
50%
66%
100%
B(1-60) 100%
Frequency
0.4
0.3
0.2
0.1
0.0
0
1
2
3
4
Foci per Cell

Supplementary Figure 4 Characterization of xNOVA-probes. Related to Figure 3. (A) Synthesis of 1C, 2x1C, or 3x1C probes carrying ATTO488 dyes. Stained DNA (SYBR Diamond) and fluorophores (ATTO488) are shown in green and red, respectively. (B) Representative confocal image of a non-repetitive region (“B”, see Figure 4A) in U2OS cells. “B” was labeled with 60 xNOVA-probes (3x1C) containing increasing labeling densities (33%, 50%, 66%, 100%). Scale bars, 5 µm. (C) xNOVA-FISH signals increase with higher labeling densities. Detected FISH intensities are displayed as boxplots. The signal was normalized by the background. Histogram of the number of foci detected in U2OS cells (n = 169). The values were normalized by the total number of cells.

	Manufacturer	Input	Costs
**NOVA-FISH**			
**1. Consumables (0.25 nmol, 100 FISH slides)**			
dNTP Mix (10 mM each)	ThermoFisher Scientific	2,5 µl	0,10 €
5-Propargylamino-dCTP-ATTO-647N (1mM)	Jena Bioscience	0,25-1,25 µl*	2,95-14,675 €*
Therminator™ DNA Polymerase	New England Biolabs	3 U	1,815 €
Monarch® PCR & DNA Cleanup Kit (250x)	New England Biolabs	1 Column	1,5 €
NucleoSpin Gel and PCR Clean-up Kit (250x)	Macherey-Nagel	1 Column	0,97 €
Thermo Scientific Lambda Exonuclease (1000 U)	ThermoFisher Scientific	10 U	1,02 €
Consumables Costs:			**8,4-20,08 €**
**2. DNA strands (for 100 Reactions)**			
**For 1 Probe:**			
Template Strand (25 nmol)	IDT	3 µg	21,66 €
Primer Strand (25 nmol)	IDT	1,5 µg	2,33 €
Total Costs:			**32.35-44.07 €**
**For 50 Probes:**			
Template Pool (50 pmol / oligo)	IDT	3 µg	106,00 €
Primer Pool (50 pmol / oligo)	IDT	1,5 µg	106,00 €
Total Costs:			**220,36-232,082 €**
**For 5 x 10 Probes:**			
Template Pool (50 pmol / oligo)	IDT	3 µg	106,00€
Primer Plate (25 nmol / oligo)	Merck	1,5 µg	220 €
Total Costs:			**367,79-426,41 €**

Supplementary Table 2 Calculated costs of NOVA-probe synthesis. Related to STAR Methods. *depending on the desired labeling density (20%, 100% fluorophore input in synthesis shown).

	Manufacturer	Yield	Costs
**oligoFISH (one-time purchase)**			
End-Labeled Secondary Probe (ATTO647N)	IDT	100 nmol***	204,97 €
**For 1 Probe:**			
Primary Probe	IDT	25 nmol	7,00 €
Total Costs:			**7,00 € (211,97 €)***
**For 50 Probes:**			
Primary Probe Pool	IDT	50 pmol/oligo	106,00 €
Total Costs:			**106,00 € (310,97 €)***
**For 5 x 10 Probes:**			
Primary Probe Plate	Merck	(25 nmol / oligo in plate)	330 €
Total Costs:			**330,00 € (534,97 €)***

Supplementary Table 3 Calculated costs of OligoFISH probes. Related to STAR Methods. *211,97 €, 310,97 €, or 534,97 € if a 3'-labeled readout probe has to be ordered.

	Manufacturer	Yield	Costs
**End-Labeled Probes (one-time purchase)**			
**For 1 Probe:**			
End-labeled Primary Probe (ATTO647N)	IDT	100 nmol*	**204,97 €**
**For 50 Probes:**			
End-labeled Primary Probe Plate (ATTO647N)	Eurofins Genomics	10 nmol / oligo	**4845 €**
**For 5 x 10 Probes:**			
End-labeled Primary Probe Plate (ATTO647N)	Eurofins Genomics	10 nmol / oligo	**4845 €**

Supplementary Table 4 Calculated costs of end-labeled probes. Related to STAR Methods.
*minimum synthesis scale

REFERENCES

S1. Pettersen, E.F., Goddard, T.D., Huang, C.C., Couch, G.S., Greenblatt, D.M., Meng, E.C., and Ferrin, T.E. (2004). UCSF Chimera--a visualization system for exploratory research and analysis. J Comput Chem *25*, 1605-1612. https://doi.org/10.1002/jcc.20084.

S2. Betz, K., Malyshev, D.A., Lavergne, T., Welte, W., Diederichs, K., Dwyer, T.J., Ordoukhanian, P., Romesberg, F.E., and Marx, A. (2012). KlenTaq polymerase replicates unnatural base pairs by inducing a Watson-Crick geometry. Nat Chem Biol *8*, 612-614. https://doi.org/10.1038/nchembio.966.

S3. Kropp, H.M., Betz, K., Wirth, J., Diederichs, K., and Marx, A. (2017). Crystal structures of ternary complexes of archaeal B-family DNA polymerases. PLoS One *12*, e0188005. https://doi.org/10.1371/journal.pone.0188005.
