# Supplementary material for: Generation of densely labeled oligonucleotides for the detection of small genomic elements
Source: Cell Rep Methods. 2024 Aug 12;4(8):100840. doi: 10.1016/j.crmeth.2024.100840 (PMC11384094; doi:10.1016/j.crmeth.2024.100840)
Supplement: Document S2. Article plus supplemental information [file mmc3.pdf]

Cell Reports
Methods

Article

Generation of densely labeled oligonucleotides for the detection of small genomic elements

Graphical abstract

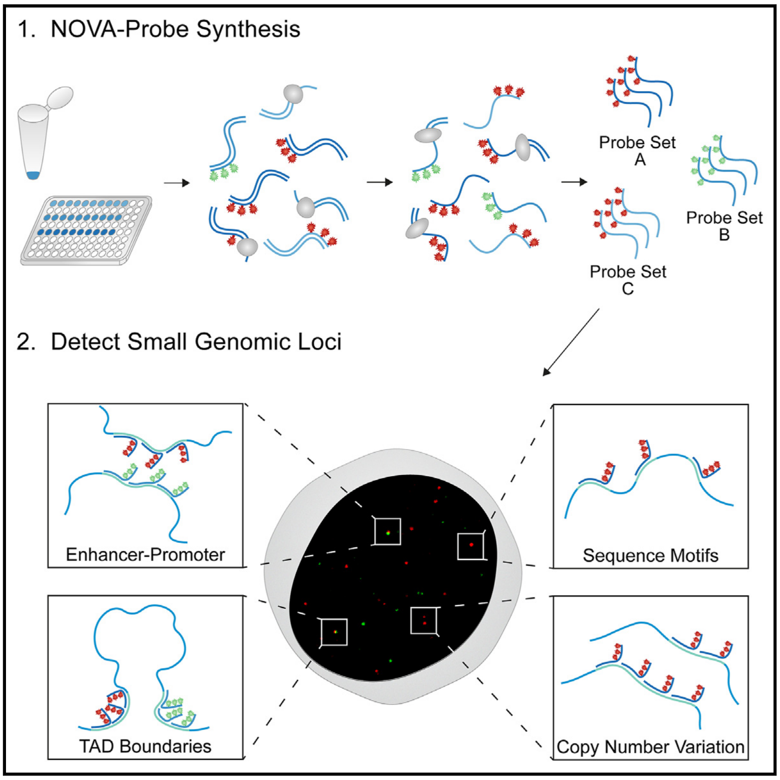

Authors

Clemens Steinek, Miguel Guirao-Ortiz, Gabriela Stumberger, ..., Thomas Carell, Hartmann Harz, Heinrich Leonhardt

Correspondence

steinek@biologie.uni-muenchen.de (C.S.),
harz@biologie.uni-muenchen.de (H.H.),
h.leonhardt@lmu.de (H.L.)

In brief

Steinek et al. develop a simple workflow for the enzymatic synthesis of densely labeled oligonucleotides. The described approach allows for free choice of fluorophores and flexible adjustment of labeling density to optimize signal detection. NOVA probes enable the detection of sub-kilobase genomic loci using confocal or super-resolution microscopy.

Highlights

- Rapid, flexible, and cost-effective synthesis of densely labeled oligonucleotides
- Systematic analysis of distance-dependent dye-dye interactions
- The NOVA workflow selectively yields labeled probe sets from complex probe pools
- NOVA-FISH enables the visualization of sub-kilobase genomic loci

Steinek et al., 2024, Cell Reports Methods *4*, 100840
August 19, 2024 © 2024 The Author(s). Published by Elsevier Inc.
https://doi.org/10.1016/j.crmeth.2024.100840

CellPress

Cell Reports Methods

CellPress
OPEN ACCESS

Article

Generation of densely labeled oligonucleotides for the detection of small genomic elements

Clemens Steinek,[1,*] Miguel Guirao-Ortiz,[1] Gabriela Stumberger,[1] Annika J. Tölke,[2] David Hörl,[1] Thomas Carell,[2] Hartmann Harz,[1,*] and Heinrich Leonhardt[1,3,*]
[1]Faculty of Biology and Center for Molecular Biosystems (BioSysM), Human Biology and BioImaging, Ludwig-Maximilians-Universität München, 81377 Munich, Germany
[2]Department of Chemistry, Ludwig-Maximilians-Universität München, 81377 Munich, Germany
[3]Lead contact
*Correspondence: steinek@biologie.uni-muenchen.de (C.S.), harz@biologie.uni-muenchen.de (H.H.), h.leonhardt@lmu.de (H.L.)
https://doi.org/10.1016/j.crmeth.2024.100840

MOTIVATION While three-dimensional chromatin conformations can be explored with fluorescence *in situ* hybridization (FISH), the visualization of small genomic loci with high spatial resolution remains challenging. For such applications, programmable oligonucleotides with high brightness are required. To further improve precision and sensitivity, secondary hybridization steps should be omitted. Here, we present a simple, quick, and inexpensive approach to generate labeled FISH probes that carry several fluorophores. Our workflow allows for the free choice of fluorophores, flexible adjustment of labeling density, and selective probe synthesis from large probe pools. With our probes, we reliably detect genomic loci below the kilobase level and examine their topological relationships.

SUMMARY

The genome contains numerous regulatory elements that may undergo complex interactions and contribute to the establishment, maintenance, and change of cellular identity. Three-dimensional genome organization can be explored with fluorescence *in situ* hybridization (FISH) at the single-cell level, but the detection of small genomic loci remains challenging. Here, we provide a rapid and simple protocol for the generation of bright FISH probes suited for the detection of small genomic elements. We systematically optimized probe design and synthesis, screened polymerases for their ability to incorporate dye-labeled nucleotides, and streamlined purification conditions to yield nanoscopy-compatible oligonucleotides with dyes in variable arrays (NOVA probes). With these probes, we detect genomic loci ranging from genome-wide repetitive regions down to non-repetitive loci below the kilobase scale. In conclusion, we introduce a simple workflow to generate densely labeled oligonucleotide pools that facilitate detection and nanoscopic measurements of small genomic elements in single cells.

INTRODUCTION

In recent years, multiple layers of mammalian genome organization ranging from preferential positions of chromosomes in the nucleus to active and inactive compartments and small-scale interactions between individual loci have been uncovered.[1–5] An intricate interplay of chromosome territories, topologically associated domains, and regulatory elements defines cellular identity in development and disease.[6–10] While current methodologies reliably probe pairwise and multi-contact DNA-DNA interactions, deciphering complex 3D chromatin organization in single cells remains challenging, particularly in the kilobase range.[11,12] Thus, there is a growing demand for increased sensitivity to detect and study DNA elements in the 3D context of individual nuclei.

The state of the art for mapping chromatin contacts is chromatin capture assays.[13–17] These methods are especially powerful, as they detect contacts within large-scale genomic regions with a resolution ranging from 1 kb down to the nucleosome level, but typically rely on population averages.[18–20] However, early efforts to probe chromatin contacts in single cells using chromatin capture assays have revealed extensive cell-to-cell variations within the same population.[21–23] Intercell variation of 3D chromatin structures has been observed in multiple imaging studies, which is consistent with the transient nature of chromatin contacts revealed by live-cell imaging.[11,12,24–30] Therefore,

This is an open access article under the CC BY license (http://creativecommons.org/licenses/by/4.0/).

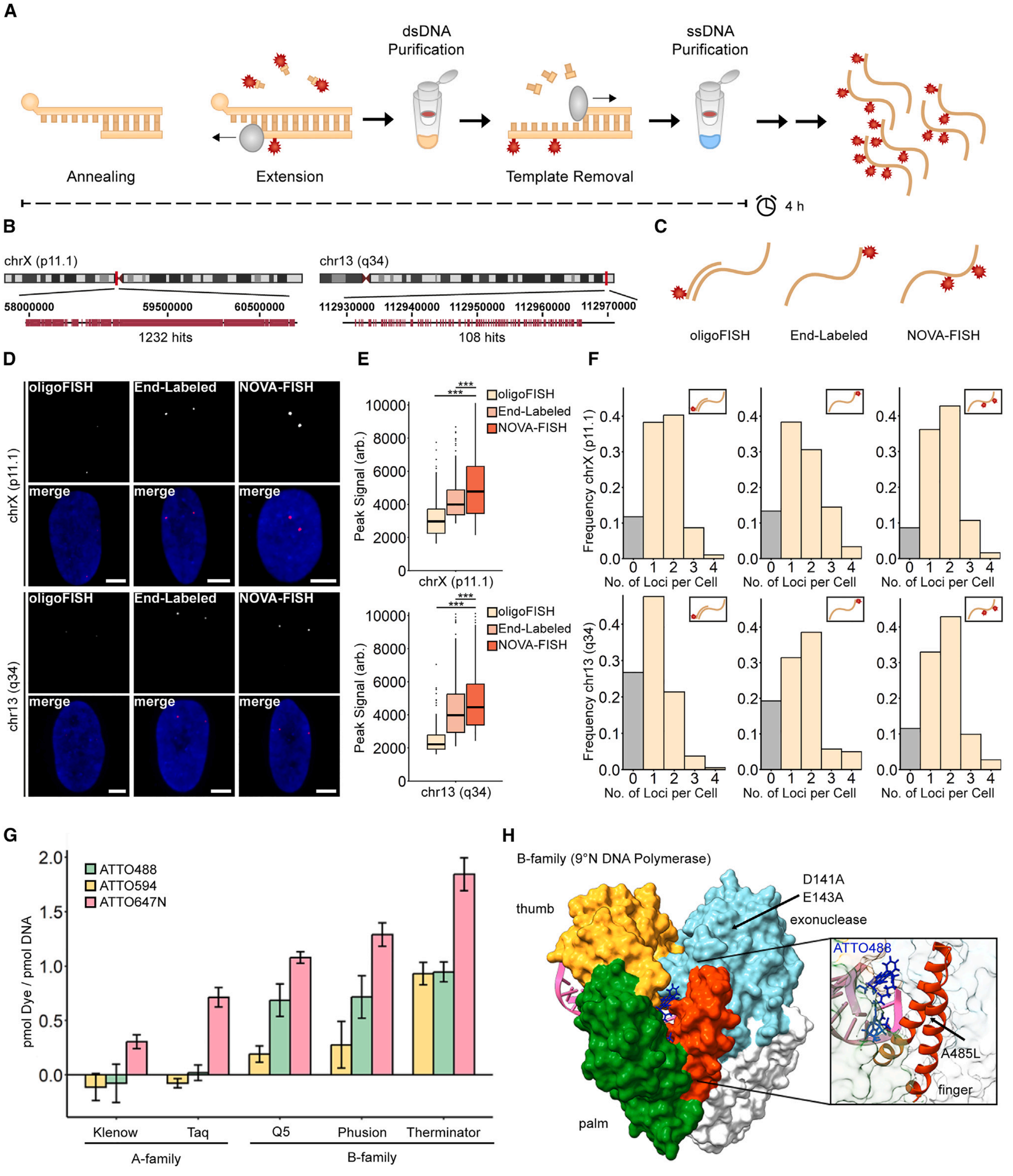

Figure 1. Generating oligonucleotides that carry multiple fluorophores

(A) Schematic workflow of the protocol. Primers are annealed to 5′ phosphorylated template strands, and dye-labeled nucleotides are incorporated in a one-step extension reaction. Template strands are then enzymatically removed, and the product is purified.

(B) Depiction of the target regions. The target regions contain a unique series of repeats (pink) in chrX (p11.1) or chr13 (q34). Human reference GRCh37/hg19 was used to retrieve coordinates.

(legend continued on next page)

CellPress
OPEN ACCESS

chromatin capture assays need to be complemented with sensitive imaging methods to comprehensively address the dynamics and function of chromatin conformations.

Since their advent, microscopy and fluorescence *in situ* hybridization (FISH) have shed light on the spatial distribution of chromatin in single cells and identified chromosomal abnormalities in malignant cells and tissues.[31–35] Although fluorescence microscopy has facilitated studies on large-scale chromatin structures, the detection and resolution of small regulatory elements with traditional FISH methods remains challenging.[24,36] In past works, FISH probes have often been generated from bacterial artificial chromosomes (BACs) or yeast artificial chromosomes (YACs) using polymerases in random priming or nick translation reactions.[37–40] However, the size of BAC or YAC probes limits the genomic resolution and is, therefore, not suitable for the detection of short regulatory DNA sequences.[41] Recent advances in synthetic DNA production and the availability of whole-genome datasets have ushered in a new era of oligonucleotide-based FISH (oligoFISH) methodologies.[28,42–47] Variations of oligoFISH utilize barcoded primary pools and fluorescent secondary readout probes to sequentially detect genomic loci. Although this approach has enabled considerable advancements in understanding chromatin architecture, the usage of single-labeled secondary probes limits the detectable target size and spatial resolution. Signal amplification has been achieved through rolling circle amplification,[48] hybridization chain reaction,[49,50] serial ligation of circular DNA (clamp-FISH[51,52]), branched DNA configurations,[53] or primer exchange reaction (SABER-FISH[54]). These techniques typically involve multiple hybridization rounds and enable detection of multiple targets, but DNA accessibility and an increased risk of non-specific amplification may complicate the visualization of small genomic elements. We hypothesized that the direct coupling of multiple fluorophores to primary oligonucleotides in combination with the elimination of secondary hybridization steps improves the signal-to-noise ratio at DNA loci of interest.

Here, we introduce a protocol to generate nanoscopy-compatible oligonucleotides with dyes in variable arrays (NOVA probes). Multiple fluorophores are attached to oligonucleotides in a one-step biochemical reaction, thereby considerably shortening the time required for probe generation. The protocol has further been optimized to allow precise control of the labeling density and does not require demanding amplification or purification steps. We applied our probes to detect a variety of genomic loci ranging from large-scale repetitive regions to sub-kilobase single loci using FISH (NOVA-FISH). Compared to previous methods, NOVA-FISH probes can efficiently be produced and allow free choice of fluorophores and flexible adjustment of labeling density to optimize signal detection in super-resolution microscopy.

RESULTS

Design and synthesis of NOVA probes

OligoFISH methods have proven valuable in visualizing genomic regions, but the necessity of multiple hybridization steps and/or the use of expensive, end-labeled probes limit their widespread application in nanoscopy. We reasoned that densely labeled oligonucleotide probe sets could be generated with an enzymatic approach in an efficient and cost-effective manner (Figure 1A). To this goal, we hybridized 5′ phosphate-labeled template strands with short primers followed by primer extension and lambda-exonuclease-mediated template degradation. We synthesized two probes that target a series of repeats on chromosome X (chrX; p11.1) or chr13 (q34) (Figure 1B). Compared with barcoded oligonucleotides and end-labeled probes, our densely labeled oligonucleotides (NOVA probes) significantly improve signal strength (Figures 1C–1E). Moreover, NOVA-FISH exhibits a significant improvement in detectability of the smaller target on chr13 (q34) ($p < 0.001$, Wilcoxon rank-sum test) but not chrX (p11.1). Therefore, NOVA probes are well suited for detecting small genomic loci (Figure 1F).

As our approach depends on the enzymatic incorporation of modified nucleotides into short primers, we compared commonly available DNA polymerases. We measured the incorporation of different dye-labeled nucleotides during extension using commonly available family A (Klenow exo-, Taq) and family B (Q5, Phusion, Therminator) DNA polymerases. Photometric measurements of synthetized probes showed that the highest labeling rates were obtained for all tested modified nucleotides with Therminator DNA polymerase (Figures 1G and S1A–S1C).

(C) Comparing three FISH strategies to tag genomic loci. While oligoFISH uses labeled readout strands for detection, end-labeled and NOVA-FISH probes carry fluorophores in their primary sequences.

(D) Representative images of both targets detected with oligoFISH, end-labeled probes, or NOVA-FISH. FISH was conducted in IMR-90 cells. Scale bars, 5 μm.

(E) NOVA-FISH yields bright FISH signals. Number of detected signals: chrX (p11.1): oligoFISH ($n = 430$), end-labeled ($n = 420$), and NOVA-FISH ($n = 548$); chr13 (q34): oligoFISH ($n = 292$), end-labeled ($n = 354$), and NOVA-FISH ($n = 413$). Datasets were tested for significance using the Wilcoxon rank-sum test with Bonferroni's correction for multiple testing (***$p < 0.001$).

(F) NOVA-FISH improves detectability in small genomic loci. Histograms depict the relative number (no.) of chrX (p11.1) or chr13 (q34) foci detected. NOVA-FISH exhibits a significant improvement in the detectability of chr13 (q34) ($p < 0.001$ for NOVA-FISH vs. oligoFISH and NOVA-FISH vs. end labeled, Wilcoxon rank-sum test). Nuclei that have been entirely imaged were included in the analysis. Number of cells analyzed: chrX (p11.1): oligoFISH ($n = 196$), end labeled ($n = 180$), and NOVA-FISH ($n = 243$); chr13 (q34): oligoFISH ($n = 187$), end labeled ($n = 157$), and NOVA-FISH ($n = 182$).

(G) Screening substrate preferences of selected DNA polymerases. Polymerases (Klenow exo-, Taq, Q5, Phusion, Therminator) incorporated dCTP-ATTO488, dCTP-ATTO594, or dCTP-ATTO647N into oligonucleotides using a 1:4 molar ratio of dye-labeled to unlabeled nucleotides. Data are represented as mean ± SD. See also Figure S1A.

(H) Crystal structure of the 9°N DNA polymerase in complex with DNA and dCTP-ATTO488. The protein is shown in white with highlighted palm (green), thumb (yellow), finger (orange), and exonuclease (cyan) domains. dCTP-ATTO488 was superimposed on the incorporated nucleotide in the complex. The magnified image depicts dCTP-ATTO488 in the binding pocket. See also Figure S2. The figure was generated with UCSF Chimera (v.1.17.3, RRID: SCR_015872) accessing 5OMV.[55,56]

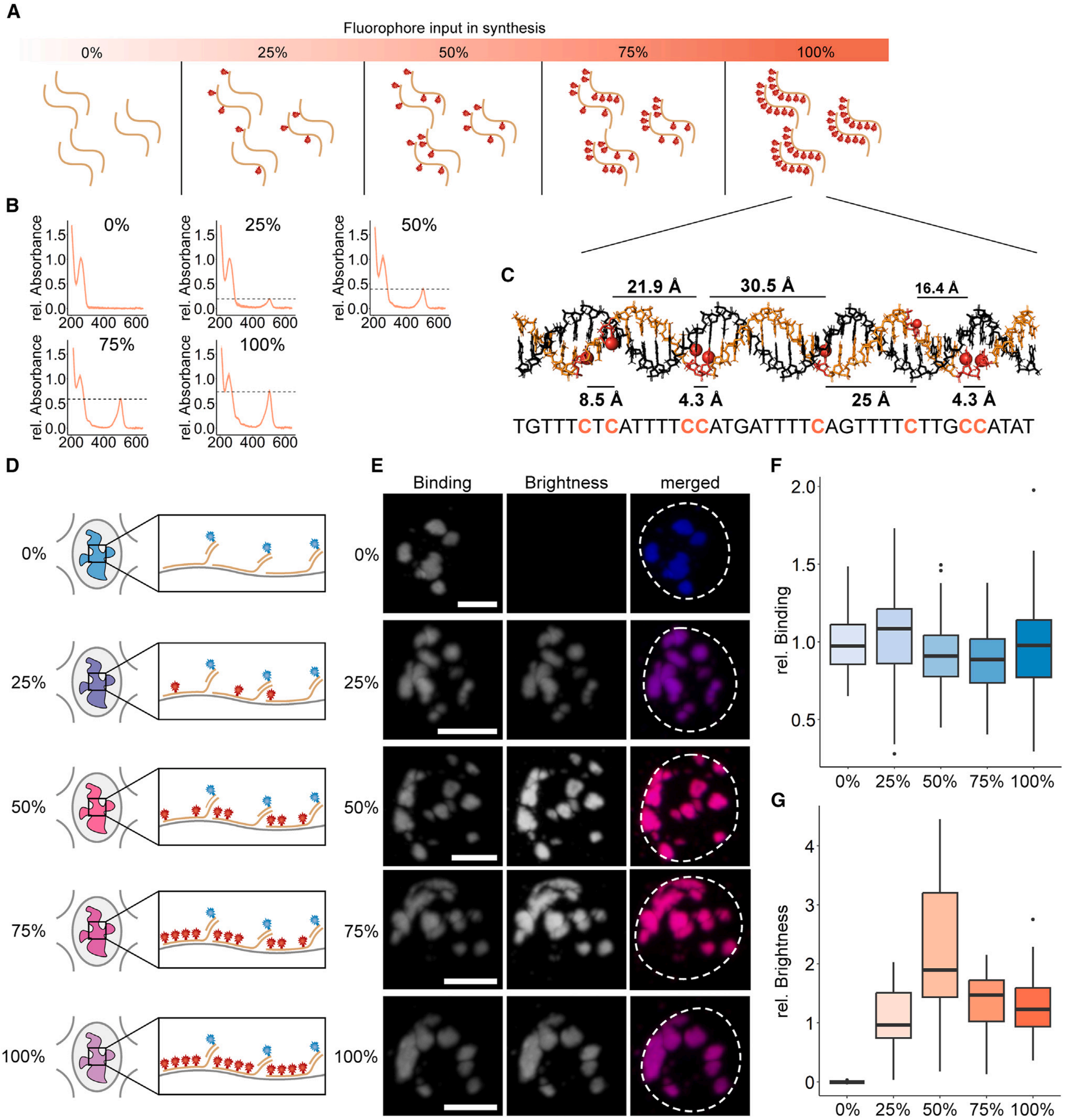

Figure 2. Binding efficiency and brightness of densely labeled probes

(A) Modulating labeling densities during NOVA probe synthesis. The labeling density is controlled through the ratio of labeled to unlabeled nucleotide (0%, 25%, 50%, 75%, 100%) in the synthesis reaction.

(B) Absorption spectra of probes with increasing labeling density. The absorbance was normalized by the absorption peak at 260 nm. The dotted lines indicate the absorption maximum of the fluorophore.

(C) Modeling fluorophore spacing in NOVA-FISH probes bound to major satellites. The B-form duplex formed by a NOVA-FISH probe (beige) and the genomic target (black) is shown. Red nucleotides indicate the locations of modified cytosines, and fluorophores are depicted as red knobs. The normal distance between neighboring fluorophores in the helix is depicted. The figure was created in Pymol v.2.5.5 (RRID: SCR_000305).[66]

(D) Assay to determine the impact of fluorophore number in the probe on hybridization efficiency. NOVA-FISH probes carrying increasing numbers of fluorophores (red) are hybridized with a locus of interest, and dye-labeled secondary strands (blue) are used as a reference.

(legend continued on next page)

Therminator DNA polymerase is a DNA polymerase that has been derived from the euryarchaeon *Thermococcus* sp. 9°N and carries mutations in its exonuclease domain (D141A, E143A) and finger domain (A485L)[57] (Figure 1H). As a result of these modifications, Therminator DNA polymerase exhibits decreased discrimination for modified nucleotides and has been used to synthesize a variety of unnatural nucleic acids.[58–61] To investigate the molecular basis for the observed variations in incorporation efficiencies among our candidates, we modeled dye-labeled nucleotides in different conformations in conjunction with finger domains of family A and B polymerases (Figure S2). We noted possible steric clashes between dye-labeled nucleotides and finger domains of family A members, whereas no such clashes were observed with family B polymerases. Using Therminator DNA polymerase, we determined that probes are robustly generated within an hour (Figure S1D). In addition, our approach allows free choice of fluorophore and flexible adjustment of labeling density (Figure S1E).

FISH probes require a high degree of purity since complementary or unlabeled strands will compete with the labeled probe during hybridization and, thus, reduce signal intensity. To remove unbound primers, free nucleotides, and enzymes, we adapted the buffer conditions to selectively yield double-stranded oligonucleotides after extension (Figures S3A–S3C). Also, unlabeled template DNA might block the synthesized probes and thereby prevent their hybridization with the locus of interest. Therefore, we have introduced phosphate groups at the 5′ ends of template strands to mark them for lambda-exonuclease-mediated degradation (Figure S3D). Using this approach, template DNA was effectively degraded within 30 min (Figure S3E). We then used ethanol-based purification to obtain the single-stranded probe (Figures S3A and S3C). This simple purification strategy yielded all NOVA probes used for microscopic measurements in this work.

After establishing a robust workflow, we assessed the number of incorporated fluorophores in NOVA probes. High-performance liquid chromatography (HPLC) analysis revealed that using a low ratio of modified to unmodified nucleotides (25%) in the synthesis reaction yields distinct elution peaks corresponding to the incorporation of increasing numbers of fluorophores (Figures S3F and S3G).

Visualizing telomere clustering below the diffraction limit

Next, we sought to utilize the brightness of NOVA probes to visualize telomeres below the diffraction limit. We tagged telomeres with telomere-specific NOVA probes and acquired images using confocal or stimulated emission depletion (STED) microscopy (Figure S1F). We observed clustered telomeres using STED microscopy, which appear as single entities in confocal images. We then applied 3D STED microscopy to gain further insights into the degree of telomere clustering (Figure S1G). Telomeres in the same cells exhibited considerable heterogeneity in their size, and clusters containing multiple telomeres were observed, consistent with previous works.[62–65] Next, we analyzed the number of detectable telomeres using confocal or STED microscopy (Figure S1G). In comparison to confocal images, STED microscopy detected, on average, 1.31 times more telomeres (±SD = 0.21), corresponding to clustered telomeres that are only resolved with super-resolution microscopy. Hence, the brightness of NOVA probes supports demanding super-resolution microscopy to visualize nuanced details of genomic loci with high optical resolution.

Dense labeling does not affect hybridization efficiency but reduces signal strength

As our workflow yields densely labeled probes, we next tested how the presence of multiple dyes in the probe affects hybridization efficiency. To address this, we generated barcoded probes with increasing labeling densities (Figures 2A–2C and S1E). These probes contain dye-labeled sequences that bind to the genome and unlabeled barcodes that hybridize with secondary probes carrying another dye. Using this approach, we can evaluate the brightness of the NOVA probe signal (green) and the relative number of probes localized at the target region (red) (Figures 2D and 2E). We found that increasing the number of dye-labeled nucleotides in the probe did not affect the number of bound probes at the locus of interest, as no notable drop in red signal was observed (Figure 2F). However, the brightness of our probes decreases at high labeling densities (Figure 2G). Consequently, densely labeled probes still bind to the region of interest, but short intermolecular distances between fluorophores impede signal strength (Figure 2C).

We next characterized the impact of dye-dye distances on probe fluorescence. We incorporated two dye molecules into overhangs of probes and increased the distances in between (1, 3, 5, 7, or 10 bases) (Figure 3A). Then, we measured the intensity of probes carrying two ATTO488 or ATTO647N molecules at the FISH spot (Figure 3B). The fluorescence of ATTO488- and ATTO647N-labeled probes increases with greater dye-dye distances (Figure 3C). Therefore, we hypothesize that distance-dependent fluorescence quenching impacts the brightness of densely labeled probes.

Establishing densely labeled probes with regularly spaced fluorophores

Our previous strategy yields labeled oligonucleotides in an efficient and cost-effective manner but depends on the occurrence of cytosines in the synthesized sequence. Therefore, we modified our workflow to generate extended probes (xNOVA probes) that carry fluorophores in a protruding sequence that does not bind to the genome (Figure 3D).[44,67] In this design, fluorophores are regularly spaced in the invariable sequence to avoid distance-dependent fluorescence quenching that might diminish

(E) Representative images of major satellites in mouse embryonic stem cells detected with NOVA-FISH probes containing increasing numbers of fluorophores. Scale bars, 5 μm.

(F) Binding efficiency is unaffected by dense labeling. The normalized intensity of dye-labeled secondary strands (blue) is depicted.

(G) Densely labeled probes exhibit a decrease in fluorescence. Related to (F). The normalized intensity of NOVA-FISH probes (red) is depicted. Number of cells analyzed: 0% (n = 149), 25% (n = 146), 50% (n = 172), 75% (n = 147), and 100% (n = 165).

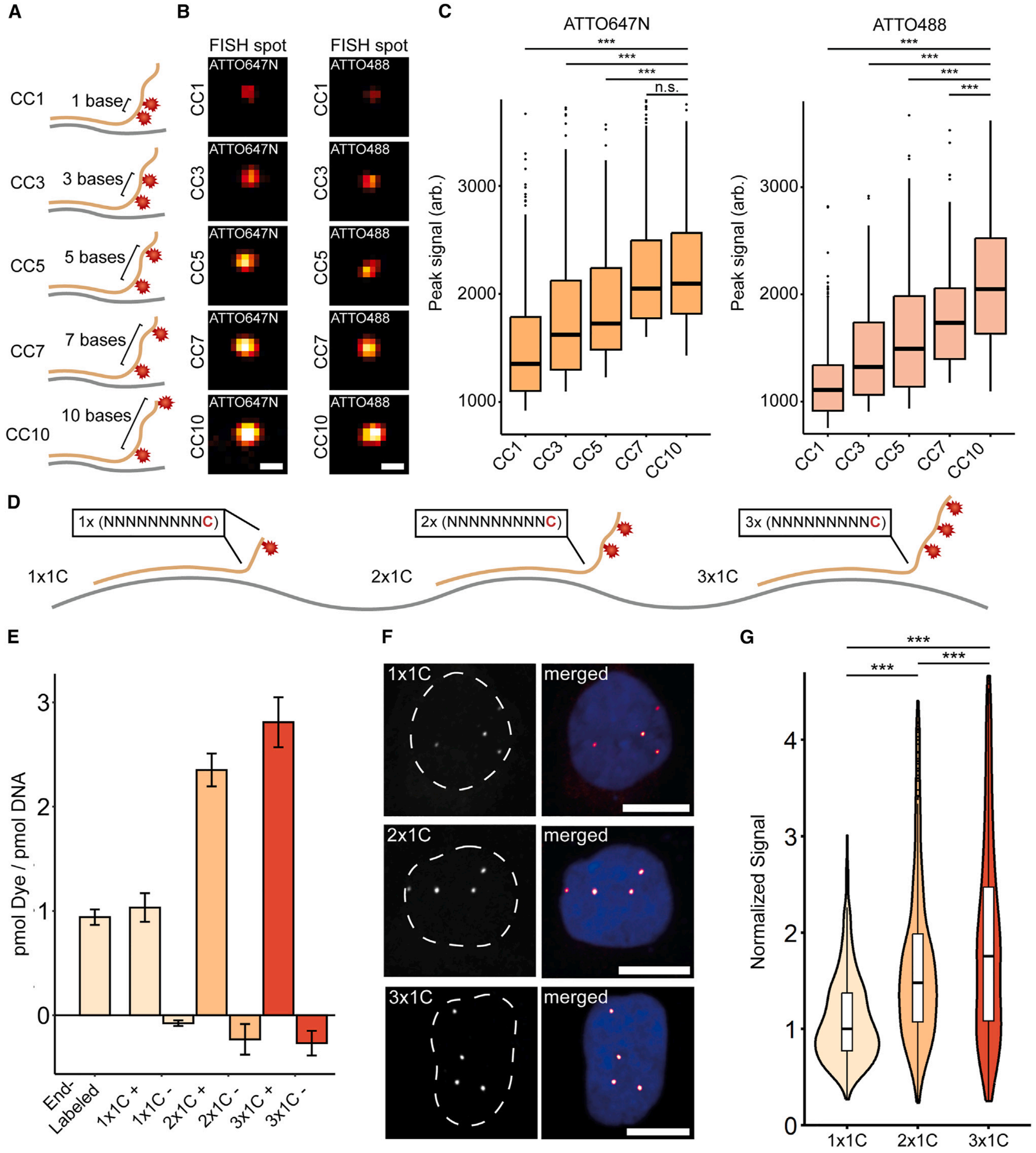

Figure 3. Designing xNOVA probes

(A) Design of probes to determine distance-dependent fluorescence quenching. NOVA probes are synthesized to carry two fluorophores with increasing distance in between (1, 3, 5, 7, or 10 bases).

(B) Representative images of chr13 (q34) targeted in IMR-90 cells. NOVA probes contain two ATTO488 or ATTO647N molecules. Scale bars, 500 nm.

(C) Dye-dye distances impact probe fluorescence. Datasets were tested for significance using the Wilcoxon rank-sum test with Bonferroni's correction for multiple testing (***$p < 0.001$).

(D) Design of extended NOVA-FISH (xNOVA) probes. xNOVA probes are extended by labeled 10-mers (NNNNNNNNNC) at their 3′ ends.

(legend continued on next page)

the specific brightness. We synthesized probes that either carried one (1 × 1C), two (2 × 1C), or three (3 × 1C) fluorophores and measured their fluorescence signals at the locus of interest (Figures 3E, 3F, and S4A). The addition of longer sequences (2 × 1C, 3 × 1C) resulted in stronger signals (Figure 3G). With this approach, we observed a steady increase in signal strength at higher labeling densities, arguing against substantial distance-dependent quenching in 3 × 1C sequences (Figures S4B and S4C).

NOVA-FISH detects non-repetitive genomic loci with kilobase resolution

Finally, we tested the limits of NOVA-FISH by detecting small non-repetitive genomic loci with nanoscale precision using STED microscopy (Figure 4A). We designed probe sets to detect non-repetitive neighboring regions on chr11 termed "A" and "B" that have been established in past works.[24] Probe sets against "A" contained 60, 50, 40, 30, 20, or 10 individual oligonucleotides, while "B" was targeted with 60 probes. The probe sets span 6.1, 4.8, 3.7, 1.7, or 0.5 kb for "A" and 4.8 kb for "B" and yield two adjacent spots (Figure 4B). A characteristic of the NOVA technology is the complete flexibility in probe synthesis, as probes can be selectively amplified from a large pool by adding appropriate primer combinations (Figure 4C). This allows the cost-effective repeated use of one oligonucleotide pool to generate probes against different target regions. Then, we targeted "A" with decreasing numbers of individual probes, maintaining the same set of probes for "B" (Figure 4D). Despite observing a decline in detection frequency with the reduced number of probes detecting "A," we were still able to detect genomic loci as small as 0.5 kb. The ratio of co-localizing spots to total number of spots is in the range of 38%–63% for A and 29%–54% for B. Furthermore, we used STED microscopy to robustly measure distances in all "A" and "B" probe pairs (Figures 4E and 4F). Thus, NOVA-FISH is a reliable tool to detect non-repetitive regions below the kilobase level.

DISCUSSION

Over the past decade, it became clear that the 3D genome organization contributes to the establishment, maintenance, and change of gene activity.[68,69] Chromatin capture assays have identified genome-wide interactions of regulatory elements and have delineated topologically associating domains (TADs).[20,70] These findings have traditionally been complemented by FISH-based imaging methods detecting entire genomes and individual chromosomes down to single genomic loci.[28,47,71] However, the optical detection of small genetic elements and the resolution of their spatial relationships at the nanoscale level remains challenging. Here, we developed a simple, rapid, flexible, and cost-effective protocol for the generation of FISH probe sets that are suited for nanoscopic measurements with kilobase resolution. In a recent study, we applied this technique to probe enhancer hijacking events upon tumorigenic translocations.[72]

Small genetic elements are ideally detected with multiple synthetic oligonucleotides that may either be directly labeled or hybridized with secondary, labeled probes. Whereas end-labeled commercial probes are expensive if large and diverse probe pools are used, enzyme-based synthesis is cost effective and flexible but requires the subsequent removal of template strands. While previously, RNA templates were reverse transcribed and subsequently degraded by RNases, we simply removed 5′ phosphorylated DNA templates using lambda exonuclease.[73] This enzymatic synthesis, including two purification steps, takes under 4 h and yields sets with hundreds of probes for less than 10 €. A detailed cost estimate of oligoFISH, end-labeled, and NOVA probes can be found in Tables S2–S4.

For enzymatic incorporation of dye-labeled nucleotides, we tested commonly available DNA polymerases. We found that B-family DNA polymerases incorporate all used modified nucleotides more effectively than A-family DNA polymerases, such as the Klenow fragment or Taq DNA polymerase. This is consistent with previous structural data of B-family polymerases, attributing their ability to incorporate dye-labeled nucleotides to a larger channel volume, the presence of B-form DNA, and phosphate backbone-mediated protein-DNA interactions.[55,74–76] Among the tested B-family polymerases, Therminator DNA polymerase, having mutations in its exonuclease domain (D141A, E143A) and finger domain (A485L), was best suited for the incorporation of dye-labeled nucleotides.[57]

As even minor FISH projects involve dozens of probes with different dye labels, we used inexpensive, commercially synthesized template pools in combination with plates of bioinformatically optimized, target-specific primers. This approach allows the flexible generation of small to large probe sets coupled with variable dyes. We demonstrate that regions as small as 500 base pairs can be detected and genomic distances of a few kilobases can be measured.

We found that the brightness of probes can be easily adjusted with the ratio of labeled to unlabeled nucleotides in the synthesis reaction. However, the brightness did not linearly increase, due to distance-dependent effects at high labeling densities. To become independent of probe-specific sequences and ensure incorporation of the same numbers of fluorescent nucleotides, we generated extended probes with overhanging, identical sequences (xNOVA). We successfully incorporated fluorophores with a spacing of ten nucleotides but note that distances down to seven nucleotides might be permissible. Our systematic analysis of distance-dependent dye-dye quenching is consistent with a previous study that measured dye-dye interactions in DNA origami.[77]

While current FISH techniques can sequentially label multiple targets, the use of end-labeled probes for secondary hybridization steps reduces signal strength. To enhance signal strength,

(E) Synthesizing xNOVA probes with specific fluorophore numbers (1 × 1C, 2 × 1C, 3 × 1C). xNOVA probes were synthesized with a 0% (−ATTO488) or 100% (+ATTO488) ratio of labeled to unlabeled nucleotides. Data are represented as mean ± SD. See also Figure S4A.

(F) Representative images of xNOVA probes detecting chr13 (q34) in U2OS cells. Scale bars, 10 μm.

(G) Quantification of xNOVA probe signals. Related to (F). The plot depicts the two brightest signals for each cell. Number of foci analyzed: 1C ($n = 513$), 2 × 1C ($n = 634$), and 3 × 1C ($n = 566$). Datasets were tested for significance using the Wilcoxon rank-sum test with Bonferroni's correction for multiple testing (***$p < 0.001$).

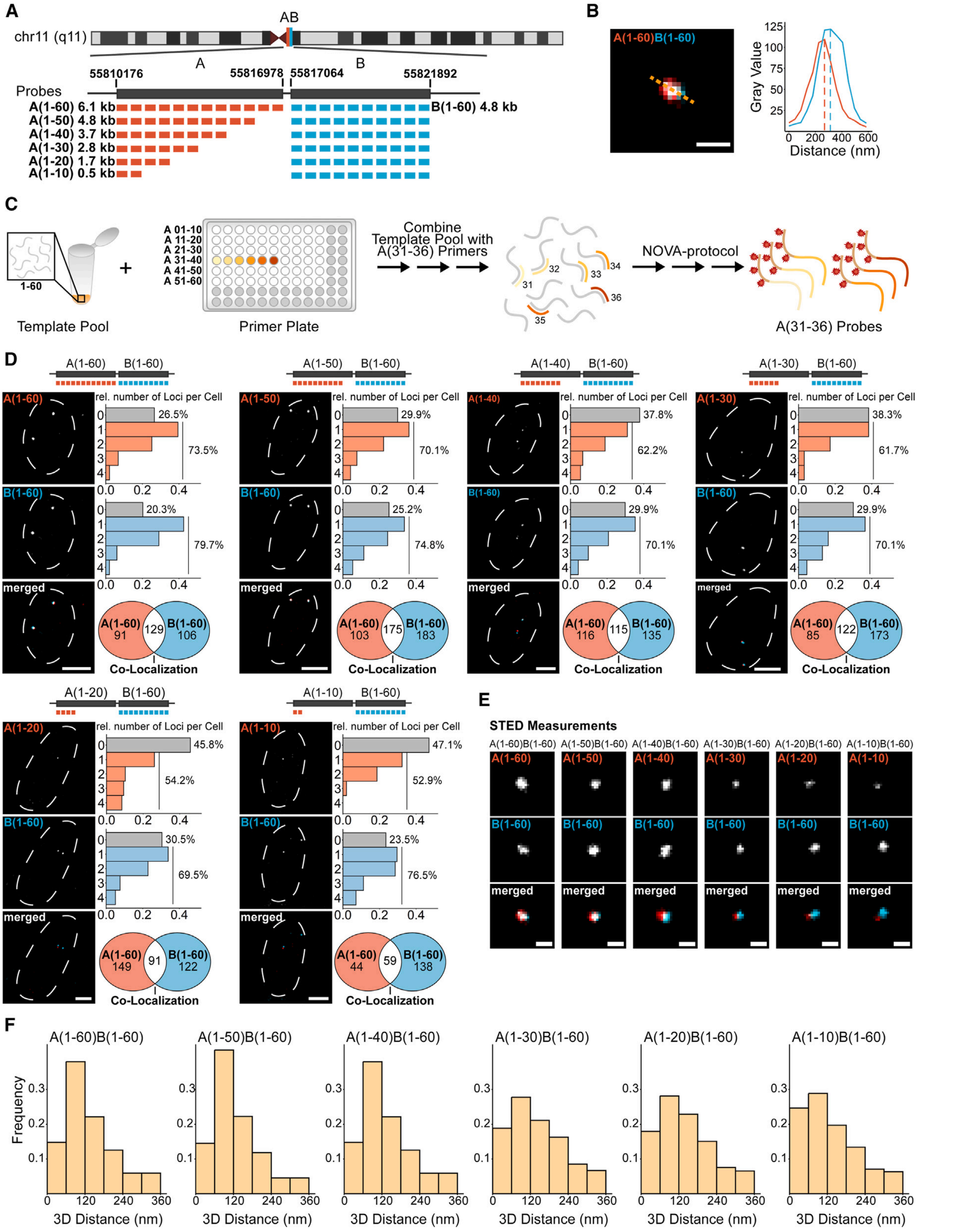

(legend on next page)

NOVA probes carrying multiple fluorophores could be employed for secondary hybridization. Moreover, we hypothesize that our workflow is suitable for applications beyond the detection of small genomic loci. Given that oligonucleotides carrying any number of desired fluorophores can be generated, opportunities in the fields of DNA-PAINT, DNA origami, or immunostainings emerge.[78,79] In summary, we present a simple, quick, and inexpensive approach to explore the spatial relationships of genetic elements governing the activity of clusters of genes.

Limitations of study

While NOVA probes enable the detection of sub-kilobase genomic loci, the number of detectable targets is currently limited by the number of distinguishable colors in the microscopy setup. Barcoded probes circumvent this limitation by sequentially binding and releasing labeled readout probes, which, however, leads to a reduction in sensitivity. NOVA probes are not compatible with multiplexed imaging techniques, as they carry fluorophores in their primary sequence. Probing the spatial relationships of a larger number of regulatory elements requires barcoded probes, which could use NOVA probes for readout.

Furthermore, NOVA probes are used for FISH experiments and therefore subject to the same general limitations of hybridization-based methods.[36,80] In particular, the same basic trade-offs between the preservation of fine structural details and hybridization penetrance apply.

STAR★METHODS

Detailed methods are provided in the online version of this paper and include the following:

- KEY RESOURCES TABLE
- RESOURCE AVAILABILITY
  - Lead contact
  - Materials availability
  - Data and code availability
- METHOD DETAILS
  - Cell culture
  - Probe design
  - NOVA-FISH Probe synthesis
  - Quality control and purification
  - Polymerase Screens
  - HPLC
  - Sample preparation and fluorescence *in situ* hybridization
  - Image acquisition
  - 3D STED microscopy of telomers using adaptive illumination
  - Automated STED microscopy for two-color NOVA-FISH
  - Image analysis
- QUANTIFICATION AND STATISTICAL ANALYSIS

SUPPLEMENTAL INFORMATION

Supplemental information can be found online at https://doi.org/10.1016/j.crmeth.2024.100840.

ACKNOWLEDGMENTS

We thank Cristina Cardoso and Irina Solovei for helpful discussions and input. This work was supported by grants from the Deutsche Forschungsgemeinschaft (SFB1064/project number 213249687 to H.L. and Priority Program SPP 2202/project number 422857584 to H.H. and H.L.) and by the BMBF in the framework of the Cluster4Future program (Cluster for Nucleic Acid Therapeutics Munich, CNATM) (project ID 03ZU1201AA to T.C. and H.L.). Microscopic images were acquired at microscopes of the Center for Advanced Light Microscopy (CALM) at LMU Munich.

AUTHOR CONTRIBUTIONS

C.S., H.L., and H.H. conceptualized the study and wrote the manuscript. C.S. synthesized NOVA probes, conducted FISH experiments, acquired microscopy images, and prepared the figures. D.H. acquired 3D STED images of telomeres and automated STED acquisition. M.G.O., G.S., and D.H. analyzed the data. A.J.T. performed HPLC purifications. H.L. and H.H. supervised the study, and H.L., H.H., and T.C. secured funding. All authors reviewed the manuscript and agreed to the published version.

DECLARATION OF INTERESTS

The authors declare no competing interests.

Received: March 12, 2024
Revised: June 16, 2024
Accepted: July 22, 2024
Published: August 12, 2024

REFERENCES

1. Cremer, T., and Cremer, M. (2010). Chromosome territories. Cold Spring Harb. Perspect. Biol. *2*, a003889. https://doi.org/10.1101/cshperspect.a003889.
2. Rao, S.S.P., Huntley, M.H., Durand, N.C., Stamenova, E.K., Bochkov, I.D., Robinson, J.T., Sanborn, A.L., Machol, I., Omer, A.D., Lander, E.S., and Aiden, E.L. (2014). A 3D map of the human genome at kilobase resolution reveals principles of chromatin looping. Cell *159*, 1665–1680. https://doi.org/10.1016/j.cell.2014.11.021.
3. Nora, E.P., Lajoie, B.R., Schulz, E.G., Giorgetti, L., Okamoto, I., Servant, N., Piolot, T., Van Berkum, N.L., Meisig, J., Sedat, J., et al. (2012). Spatial

Figure 4. Using xNOVA probes to detect non-repetitive loci at kilobase scale

(A) Depiction of the target regions. Two adjacent non-repetitive genomic regions on chr11 (hg19, chr11: 55,810,176–55,816,978, hg19, chr11: 55,817,064–55,821,892) were targeted with 60 xNOVA probes spanning 6.1 or 4.8 kb. In successive experiments, the number of xNOVA probes targeting A was gradually reduced from 60 to 10 probes. 60 xNOVA probes targeting B were used as a reference.
(B) Representative STED images of regions “A” and “B” in two colors. A(1–60) was tagged with ATTO594, and B(1–60) harbored ATTO647N. Scale bar, 500 nm.
(C) Schematic of selective xNOVA probe synthesis. Probe sets are selectively synthesized through the combination of region-specific primers with common template pools.
(D) Detecting kilobase genomic loci using confocal microscopy. 60 probes detecting B were paired with probe sets comprising 60 to 10 individual probes for A. Histograms depict the relative number of detected loci per cell. Venn diagrams depict the number of detected single and co-localizing signals. Scale bars, 5 μm. Number of cells analyzed: A(1–60)B(1–60): $n = 151$; A(1–50)B(1–60): $n = 153$; A(1–40)B(1–60): $n = 172$; A(1–30)B(1–60): $n = 168$; A(1–20)B(1–60): $n = 124$; A(1–10)B(1–60): $n = 101$.
(E) Representative z projections of A paired with B in K562 cells. Scale bars, 200 nm.
(F) 3D distance of A and B. Number of analyzed spot pairs: 1–60: $n = 550$; 1–50: $n = 388$; 1–40: $n = 317$; 1–30: $n = 329$; 1–20: $n = 445$; 1–10: $n = 226$.

partitioning of the regulatory landscape of the X-inactivation centre. Nature *485*, 381–385. https://doi.org/10.1038/nature11049.

4. Jeong, Y., El-Jaick, K., Roessler, E., Muenke, M., and Epstein, D.J. (2006). A functional screen for sonic hedgehog regulatory elements across a 1 Mb interval identifies long-range ventral forebrain enhancers. Development *133*, 761–772. https://doi.org/10.1242/dev.02239.
5. Pombo, A., and Dillon, N. (2015). Three-dimensional genome architecture: players and mechanisms. Nat. Rev. Mol. Cell Biol. *16*, 245–257. https://doi.org/10.1038/nrm3965.
6. Medrano-Fernandez, A., and Barco, A. (2016). Nuclear organization and 3D chromatin architecture in cognition and neuropsychiatric disorders. Mol. Brain *9*, 83. https://doi.org/10.1186/s13041-016-0263-x.
7. Lupiáñez, D.G., Kraft, K., Heinrich, V., Krawitz, P., Brancati, F., Klopocki, E., Horn, D., Kayserili, H., Opitz, J.M., Laxova, R., et al. (2015). Disruptions of topological chromatin domains cause pathogenic rewiring of gene-enhancer interactions. Cell *161*, 1012–1025. https://doi.org/10.1016/j.cell.2015.04.004.
8. Hnisz, D., Weintraub, A.S., Day, D.S., Valton, A.L., Bak, R.O., Li, C.H., Goldmann, J., Lajoie, B.R., Fan, Z.P., Sigova, A.A., et al. (2016). Activation of proto-oncogenes by disruption of chromosome neighborhoods. Science *351*, 1454–1458. https://doi.org/10.1126/science.aad9024.
9. Schuijers, J., Manteiga, J.C., Weintraub, A.S., Day, D.S., Zamudio, A.V., Hnisz, D., Lee, T.I., and Young, R.A. (2018). Transcriptional Dysregulation of MYC Reveals Common Enhancer-Docking Mechanism. Cell Rep. *23*, 349–360. https://doi.org/10.1016/j.celrep.2018.03.056.
10. Dixon, J.R., Jung, I., Selvaraj, S., Shen, Y., Antosiewicz-Bourget, J.E., Lee, A.Y., Ye, Z., Kim, A., Rajagopal, N., Xie, W., et al. (2015). Chromatin architecture reorganization during stem cell differentiation. Nature *518*, 331–336. https://doi.org/10.1038/nature14222.
11. Mateo, L.J., Murphy, S.E., Hafner, A., Cinquini, I.S., Walker, C.A., and Boettiger, A.N. (2019). Visualizing DNA folding and RNA in embryos at single-cell resolution. Nature *568*, 49–54. https://doi.org/10.1038/s41586-019-1035-4.
12. Gizzi, A.M.C., Cattoni, D.I., Fiche, J.-B., Espinola, S.M., Gurgo, J., Messina, O., Houbron, C., Ogiyama, Y., Papadopoulos, G.L., and Cavalli, G. (2019). Microscopy-based chromosome conformation capture enables simultaneous visualization of genome organization and transcription in intact organisms. Mol. Cell *74*, 212–222. https://doi.org/10.1016/j.molcel.2019.01.011.
13. Simonis, M., Klous, P., Splinter, E., Moshkin, Y., Willemsen, R., de Wit, E., van Steensel, B., and de Laat, W. (2006). Nuclear organization of active and inactive chromatin domains uncovered by chromosome conformation capture-on-chip (4C). Nat. Genet. *38*, 1348–1354. https://doi.org/10.1038/ng1896.
14. Dekker, J., Rippe, K., Dekker, M., and Kleckner, N. (2002). Capturing chromosome conformation. Science *295*, 1306–1311. https://doi.org/10.1126/science.1067799.
15. Lieberman-Aiden, E., van Berkum, N.L., Williams, L., Imakaev, M., Ragoczy, T., Telling, A., Amit, I., Lajoie, B.R., Sabo, P.J., Dorschner, M.O., et al. (2009). Comprehensive mapping of long-range interactions reveals folding principles of the human genome. Science *326*, 289–293. https://doi.org/10.1126/science.1181369.
16. Krietenstein, N., Abraham, S., Venev, S.V., Abdennur, N., Gibcus, J., Hsieh, T.H.S., Parsi, K.M., Yang, L., Maehr, R., Mirny, L.A., et al. (2020). Ultrastructural Details of Mammalian Chromosome Architecture. Mol. Cell *78*, 554–565.e7. https://doi.org/10.1016/j.molcel.2020.03.003.
17. Dixon, J.R., Selvaraj, S., Yue, F., Kim, A., Li, Y., Shen, Y., Hu, M., Liu, J.S., and Ren, B. (2012). Topological domains in mammalian genomes identified by analysis of chromatin interactions. Nature *485*, 376–380. https://doi.org/10.1038/nature11082.
18. Jerkovic, I., and Cavalli, G. (2021). Understanding 3D genome organization by multidisciplinary methods. Nat. Rev. Mol. Cell Biol. *22*, 511–528. https://doi.org/10.1038/s41580-021-00362-w.
19. Goel, V.Y., and Hansen, A.S. (2021). The macro and micro of chromosome conformation capture. Wiley Interdiscip. Rev. Dev. Biol. *10*, e395. https://doi.org/10.1002/wdev.395.
20. McCord, R.P., Kaplan, N., and Giorgetti, L. (2020). Chromosome Conformation Capture and Beyond: Toward an Integrative View of Chromosome Structure and Function. Mol. Cell *77*, 688–708. https://doi.org/10.1016/j.molcel.2019.12.021.
21. Nagano, T., Lubling, Y., Stevens, T.J., Schoenfelder, S., Yaffe, E., Dean, W., Laue, E.D., Tanay, A., and Fraser, P. (2013). Single-cell Hi-C reveals cell-to-cell variability in chromosome structure. Nature *502*, 59–64. https://doi.org/10.1038/nature12593.
22. Flyamer, I.M., Gassler, J., Imakaev, M., Brandão, H.B., Ulianov, S.V., Abdennur, N., Razin, S.V., Mirny, L.A., and Tachibana-Konwalski, K. (2017). Single-nucleus Hi-C reveals unique chromatin reorganization at oocyte-to-zygote transition. Nature *544*, 110–114. https://doi.org/10.1038/nature21711.
23. Stevens, T.J., Lando, D., Basu, S., Atkinson, L.P., Cao, Y., Lee, S.F., Leeb, M., Wohlfahrt, K.J., Boucher, W., O'Shaughnessy-Kirwan, A., et al. (2017). 3D structures of individual mammalian genomes studied by single-cell Hi-C. Nature *544*, 59–64. https://doi.org/10.1038/nature21429.
24. Brandstetter, K., Zülske, T., Ragoczy, T., Hörl, D., Guirao-Ortiz, M., Steinek, C., Barnes, T., Stumberger, G., Schwach, J., Haugen, E., et al. (2022). Differences in nanoscale organization of regulatory active and inactive human chromatin. Biophys. J. *121*, 977–990. https://doi.org/10.1016/j.bpj.2022.02.009.
25. Levi, V., Ruan, Q., Plutz, M., Belmont, A.S., and Gratton, E. (2005). Chromatin dynamics in interphase cells revealed by tracking in a two-photon excitation microscope. Biophys. J. *89*, 4275–4285. https://doi.org/10.1529/biophysj.105.066670.
26. Gabriele, M., Brandão, H.B., Grosse-Holz, S., Jha, A., Dailey, G.M., Cattoglio, C., Hsieh, T.-H.S., Mirny, L., Zechner, C., and Hansen, A.S. (2022). Dynamics of CTCF-and cohesin-mediated chromatin looping revealed by live-cell imaging. Science *376*, 496–501. https://doi.org/10.1126/science.abn6583.
27. Giorgetti, L., Galupa, R., Nora, E.P., Piolot, T., Lam, F., Dekker, J., Tiana, G., and Heard, E. (2014). Predictive polymer modeling reveals coupled fluctuations in chromosome conformation and transcription. Cell *157*, 950–963. https://doi.org/10.1016/j.cell.2014.03.025.
28. Bintu, B., Mateo, L.J., Su, J.H., Sinnott-Armstrong, N.A., Parker, M., Kinrot, S., Yamaya, K., Boettiger, A.N., and Zhuang, X. (2018). Super-resolution chromatin tracing reveals domains and cooperative interactions in single cells. Science *362*, eaau1783. https://doi.org/10.1126/science.aau1783.
29. Finn, E.H., Pegoraro, G., Brandao, H.B., Valton, A.L., Oomen, M.E., Dekker, J., Mirny, L., and Misteli, T. (2019). Extensive Heterogeneity and Intrinsic Variation in Spatial Genome Organization. Cell *176*, 1502–1515. https://doi.org/10.1016/j.cell.2019.01.020.
30. Cattoni, D.I., Cardozo Gizzi, A.M., Georgieva, M., Di Stefano, M., Valeri, A., Chamousset, D., Houbron, C., Déjardin, S., Fiche, J.B., González, I., et al. (2017). Single-cell absolute contact probability detection reveals chromosomes are organized by multiple low-frequency yet specific interactions. Nat. Commun. *8*, 1753. https://doi.org/10.1038/s41467-017-01962-x.
31. Bolzer, A., Kreth, G., Solovei, I., Koehler, D., Saracoglu, K., Fauth, C., Müller, S., Eils, R., Cremer, C., Speicher, M.R., and Cremer, T. (2005). Three-dimensional maps of all chromosomes in human male fibroblast nuclei and prometaphase rosettes. PLoS Biol. *3*, e157. https://doi.org/10.1371/journal.pbio.0030157.
32. Kallioniemi, O.P., Kallioniemi, A., Kurisu, W., Thor, A., Chen, L.C., Smith, H.S., Waldman, F.M., Pinkel, D., and Gray, J.W. (1992). ERBB2 amplification in breast cancer analyzed by fluorescence in situ hybridization. Proc. Natl. Acad. Sci. USA *89*, 5321–5325. https://doi.org/10.1073/pnas.89.12.5321.

33. Cui, C., Shu, W., and Li, P. (2016). Fluorescence In situ Hybridization: Cell-Based Genetic Diagnostic and Research Applications. Front. Cell Dev. Biol. *4*, 89. https://doi.org/10.3389/fcell.2016.00089.

34. Chrzanowska, N.M., Kowalewski, J., and Lewandowska, M.A. (2020). Use of Fluorescence In Situ Hybridization (FISH) in Diagnosis and Tailored Therapies in Solid Tumors. Molecules *25*, 1864. https://doi.org/10.3390/molecules25081864.

35. Pardue, M.L., and Gall, J.G. (1969). Molecular hybridization of radioactive DNA to the DNA of cytological preparations. Proc. Natl. Acad. Sci. USA *64*, 600–604. https://doi.org/10.1073/pnas.64.2.600.

36. Markaki, Y., Smeets, D., Fiedler, S., Schmid, V.J., Schermelleh, L., Cremer, T., and Cremer, M. (2012). The potential of 3D-FISH and super-resolution structured illumination microscopy for studies of 3D nuclear architecture: 3D structured illumination microscopy of defined chromosomal structures visualized by 3D (immuno)-FISH opens new perspectives for studies of nuclear architecture. Bioessays *34*, 412–426. https://doi.org/10.1002/bies.201100176.

37. Wiegant, J., Ried, T., Nederlof, P.M., van der Ploeg, M., Tanke, H.J., and Raap, A.K. (1991). In situ hybridization with fluoresceinated DNA. Nucleic Acids Res. *19*, 3237–3241. https://doi.org/10.1093/nar/19.12.3237.

38. Itzkovitz, S., and van Oudenaarden, A. (2011). Validating transcripts with probes and imaging technology. Nat. Methods *8*, S12–S19. https://doi.org/10.1038/nmeth.1573.

39. Shizuya, H., Birren, B., Kim, U.J., Mancino, V., Slepak, T., Tachiiri, Y., and Simon, M. (1992). Cloning and stable maintenance of 300-kilobase-pair fragments of human DNA in Escherichia coli using an F-factor-based vector. Proc. Natl. Acad. Sci. USA *89*, 8794–8797. https://doi.org/10.1073/pnas.89.18.8794.

40. Burke, D.T., Carle, G.F., and Olson, M.V. (1987). Cloning of large segments of exogenous DNA into yeast by means of artificial chromosome vectors. Science *236*, 806–812. https://doi.org/10.1126/science.3033825.

41. Boettiger, A., and Murphy, S. (2020). Advances in chromatin imaging at kilobase-scale resolution. TiG *36*, 273–287. https://doi.org/10.1016/j.tig.2019.12.010.

42. Huber, D., Voith von Voithenberg, L., and Kaigala, G. (2018). Fluorescence in situ hybridization (FISH): history, limitations and what to expect from micro-scale FISH? Micro Nano Eng. *1*, 15–24. https://doi.org/10.1016/j.mne.2018.10.006.

43. Raj, A., van den Bogaard, P., Rifkin, S.A., van Oudenaarden, A., and Tyagi, S. (2008). Imaging individual mRNA molecules using multiple singly labeled probes. Nat. Methods *5*, 877–879. https://doi.org/10.1038/nmeth.1253.

44. Beliveau, B.J., Joyce, E.F., Apostolopoulos, N., Yilmaz, F., Fonseka, C.Y., McCole, R.B., Chang, Y., Li, J.B., Senaratne, T.N., Williams, B.R., et al. (2012). Versatile design and synthesis platform for visualizing genomes with Oligopaint FISH probes. Proc. Natl. Acad. Sci. USA *109*, 21301–21306. https://doi.org/10.1073/pnas.1213818110.

45. Su, J.H., Zheng, P., Kinrot, S.S., Bintu, B., and Zhuang, X. (2020). Genome-Scale Imaging of the 3D Organization and Transcriptional Activity of Chromatin. Cell *182*, 1641–1659.e26. https://doi.org/10.1016/j.cell.2020.07.032.

46. Takei, Y., Zheng, S., Yun, J., Shah, S., Pierson, N., White, J., Schindler, S., Tischbirek, C.H., Yuan, G.C., and Cai, L. (2021). Single-cell nuclear architecture across cell types in the mouse brain. Science *374*, 586–594. https://doi.org/10.1126/science.abj1966.

47. Szabo, Q., Donjon, A., Jerković, I., Papadopoulos, G.L., Cheutin, T., Bonev, B., Nora, E.P., Bruneau, B.G., Bantignies, F., and Cavalli, G. (2020). Regulation of single-cell genome organization into TADs and chromatin nanodomains. Nat. Genet. *52*, 1151–1157. https://doi.org/10.1038/s41588-020-00716-8.

48. Lizardi, P.M., Huang, X., Zhu, Z., Bray-Ward, P., Thomas, D.C., and Ward, D.C. (1998). Mutation detection and single-molecule counting using isothermal rolling-circle amplification. Nat. Genet. *19*, 225–232. https://doi.org/10.1038/898.

49. Dirks, R.M., and Pierce, N.A. (2004). Triggered amplification by hybridization chain reaction. Proc. Natl. Acad. Sci. USA *101*, 15275–15278. https://doi.org/10.1073/pnas.0407024101.

50. Choi, H.M.T., Beck, V.A., and Pierce, N.A. (2014). Next-generation in situ hybridization chain reaction: higher gain, lower cost, greater durability. ACS Nano *8*, 4284–4294. https://doi.org/10.1021/nn405717p.

51. Rouhanifard, S.H., Mellis, I.A., Dunagin, M., Bayatpour, S., Jiang, C.L., Dardani, I., Symmons, O., Emert, B., Torre, E., Cote, A., et al. (2019). Amendments: Author Correction: ClampFISH detects individual nucleic acid molecules using click chemistry-based amplification. Nat. Biotechnol. *37*, 102. https://doi.org/10.1038/nbt0119-102b.

52. Dardani, I., Emert, B.L., Goyal, Y., Jiang, C.L., Kaur, A., Lee, J., Rouhanifard, S.H., Alicea, G.M., Fane, M.E., Xiao, M., et al. (2022). ClampFISH 2.0 enables rapid, scalable amplified RNA detection in situ. Nat. Methods *19*, 1403–1410. https://doi.org/10.1038/s41592-022-01653-6.

53. Xie, F., Timme, K.A., and Wood, J.R. (2018). Using Single Molecule mRNA Fluorescent in Situ Hybridization (RNA-FISH) to Quantify mRNAs in Individual Murine Oocytes and Embryos. Sci. Rep. *8*, 7930. https://doi.org/10.1038/s41598-018-26345-0.

54. Kishi, J.Y., Lapan, S.W., Beliveau, B.J., West, E.R., Zhu, A., Sasaki, H.M., Saka, S.K., Wang, Y., Cepko, C.L., and Yin, P. (2019). SABER amplifies FISH: enhanced multiplexed imaging of RNA and DNA in cells and tissues. Nat. Methods *16*, 533–544. https://doi.org/10.1038/s41592-019-0404-0.

55. Kropp, H.M., Betz, K., Wirth, J., Diederichs, K., and Marx, A. (2017). Crystal structures of ternary complexes of archaeal B-family DNA polymerases. PLoS One *12*, e0188005. https://doi.org/10.1371/journal.pone.0188005.

56. Pettersen, E.F., Goddard, T.D., Huang, C.C., Couch, G.S., Greenblatt, D.M., Meng, E.C., and Ferrin, T.E. (2004). UCSF Chimera–a visualization system for exploratory research and analysis. J. Comput. Chem. *25*, 1605–1612. https://doi.org/10.1002/jcc.20084.

57. Gardner, A.F., Jackson, K.M., Boyle, M.M., Buss, J.A., Potapov, V., Gehring, A.M., Zatopek, K.M., Corrêa, I.R., Jr., Ong, J.L., and Jack, W.E. (2019). Therminator DNA Polymerase: Modified Nucleotides and Unnatural Substrates. Front. Mol. Biosci. *6*, 28. https://doi.org/10.3389/fmolb.2019.00028.

58. Ichida, J.K., Horhota, A., Zou, K., McLaughlin, L.W., and Szostak, J.W. (2005). High fidelity TNA synthesis by Therminator polymerase. Nucleic Acids Res. *33*, 5219–5225. https://doi.org/10.1093/nar/gki840.

59. Renders, M., Emmerechts, G., Rozenski, J., Krecmerová, M., Holý, A., and Herdewijn, P. (2007). Enzymatic synthesis of phosphonomethyl oligonucleotides by therminator polymerase. Angew. Chem., Int. Ed. Engl. *46*, 2501–2504. https://doi.org/10.1002/anie.200603435.

60. Mohsen, M.G., Ji, D., and Kool, E.T. (2019). Polymerase synthesis of four-base DNA from two stable dimeric nucleotides. Nucleic Acids Res. *47*, 9495–9501. https://doi.org/10.1093/nar/gkz741.

61. Gardner, A.F., and Jack, W.E. (2002). Acyclic and dideoxy terminator preferences denote divergent sugar recognition by archaeon and Taq DNA polymerases. Nucleic Acids Res. *30*, 605–613. https://doi.org/10.1093/nar/30.2.605.

62. Jegou, T., Chung, I., Heuvelman, G., Wachsmuth, M., Görisch, S.M., Greulich-Bode, K.M., Boukamp, P., Lichter, P., and Rippe, K. (2009). Dynamics of telomeres and promyelocytic leukemia nuclear bodies in a telomerase-negative human cell line. Mol. Biol. Cell *20*, 2070–2082. https://doi.org/10.1091/mbc.e08-02-0108.

63. Crabbe, L., Cesare, A.J., Kasuboski, J.M., Fitzpatrick, J.A.J., and Karlseder, J. (2012). Human telomeres are tethered to the nuclear envelope during postmitotic nuclear assembly. Cell Rep. *2*, 1521–1529. https://doi.org/10.1016/j.celrep.2012.11.019.

64. Chuang, T.C.Y., Moshir, S., Garini, Y., Chuang, A.Y.C., Young, I.T., Vermolen, B., van den Doel, R., Mougey, V., Perrin, M., Braun, M., et al.

(2004). The three-dimensional organization of telomeres in the nucleus of mammalian cells. BMC Biol. *2*, 12. https://doi.org/10.1186/1741-7007-2-12.

65. Adam, N., Degelman, E., Briggs, S., Wazen, R.M., Colarusso, P., Riabowol, K., and Beattie, T. (2019). Telomere analysis using 3D fluorescence microscopy suggests mammalian telomere clustering in hTERT-immortalized Hs68 fibroblasts. Commun. Biol. *2*, 451. https://doi.org/10.1038/s42003-019-0692-z.
66. DeLano, W.L. (2002). Pymol: an open-source molecular graphics tool. CCP4 Newsletter Pro. Crystallogr. *40*, 82–92.
67. Beliveau, B.J., Boettiger, A.N., Avendaño, M.S., Jungmann, R., McCole, R.B., Joyce, E.F., Kim-Kiselak, C., Bantignies, F., Fonseka, C.Y., Erceg, J., et al. (2015). Single-molecule super-resolution imaging of chromosomes and in situ haplotype visualization using Oligopaint FISH probes. Nat. Commun. *6*, 7147. https://doi.org/10.1038/ncomms8147.
68. Willemin, A., Szabó, D., and Pombo, A. (2024). Epigenetic regulatory layers in the 3D nucleus. Mol. Cell *84*, 415–428. https://doi.org/10.1016/j.molcel.2023.12.032.
69. Zheng, H., and Xie, W. (2019). The role of 3D genome organization in development and cell differentiation. Nat. Rev. Mol. Cell Biol. *20*, 535–550. https://doi.org/10.1038/s41580-019-0132-4.
70. Sati, S., and Cavalli, G. (2017). Chromosome conformation capture technologies and their impact in understanding genome function. Chromosoma *126*, 33–44. https://doi.org/10.1007/s00412-016-0593-6.
71. Peng, T., Hou, Y., Meng, H., Cao, Y., Wang, X., Jia, L., Chen, Q., Zheng, Y., Sun, Y., Chen, H., et al. (2023). Mapping nucleolus-associated chromatin interactions using nucleolus Hi-C reveals pattern of heterochromatin interactions. Nat. Commun. *14*, 350. https://doi.org/10.1038/s41467-023-36021-1.
72. Weichenhan, D., Riedel, A., Sollier, E., Toprak, U.H., Hey, J., Breuer, K., Wierzbinska, J.A., Touzart, A., Lutsik, P., Bähr, M., et al. (2023). Altered enhancer-promoter interaction leads to MNX1 expression in pediatric acute myeloid leukemia with t (7; 12)(q36; p13). Preprint at bioRxiv. https://doi.org/10.1101/2023.09.13.557546.
73. Beliveau, B.J., Boettiger, A.N., Nir, G., Bintu, B., Yin, P., Zhuang, X., and Wu, C.T. (2017). In Situ Super-Resolution Imaging of Genomic DNA with OligoSTORM and OligoDNA-PAINT. Methods Mol. Biol. *1663*, 231–252. https://doi.org/10.1007/978-1-4939-7265-4_19.
74. Hottin, A., and Marx, A. (2016). Structural insights into the processing of nucleobase-modified nucleotides by DNA polymerases. Acc. Chem. Res. *49*, 418–427. https://doi.org/10.1021/acs.accounts.5b00544.
75. Tasara, T., Angerer, B., Damond, M., Winter, H., Dörhöfer, S., Hübscher, U., and Amacker, M. (2003). Incorporation of reporter molecule-labeled nucleotides by DNA polymerases. II. High-density labeling of natural DNA. Nucleic Acids Res. *31*, 2636–2646. https://doi.org/10.1093/nar/gkg371.
76. Kropp, H.M., Diederichs, K., and Marx, A. (2019). The Structure of an archaeal B-family DNA polymerase in complex with a chemically modified nucleotide. Angew. Chem., Int. Ed. Engl. *58*, 5457–5461. https://doi.org/10.1002/anie.201900315.
77. Schröder, T., Scheible, M.B., Steiner, F., Vogelsang, J., and Tinnefeld, P. (2019). Interchromophoric Interactions Determine the Maximum Brightness Density in DNA Origami Structures. Nano Lett. *19*, 1275–1281. https://doi.org/10.1021/acs.nanolett.8b04845.
78. Jungmann, R., Avendaño, M.S., Woehrstein, J.B., Dai, M., Shih, W.M., and Yin, P. (2014). Multiplexed 3D cellular super-resolution imaging with DNA-PAINT and Exchange-PAINT. Nat. Methods *11*, 313–318. https://doi.org/10.1038/nmeth.2835.
79. Schnitzbauer, J., Strauss, M.T., Schlichthaerle, T., Schueder, F., and Jungmann, R. (2017). Super-resolution microscopy with DNA-PAINT. Nat. Protoc. *12*, 1198–1228. https://doi.org/10.1038/nprot.2017.024.
80. Solovei, I., Cavallo, A., Schermelleh, L., Jaunin, F., Scasselati, C., Cmarko, D., Cremer, C., Fakan, S., and Cremer, T. (2002). Spatial preservation of nuclear chromatin architecture during three-dimensional fluorescence in situ hybridization (3D-FISH). Exp. Cell Res. *276*, 10–23. https://doi.org/10.1006/excr.2002.5513.
81. Anton, T., Bultmann, S., Leonhardt, H., and Markaki, Y. (2014). Visualization of specific DNA sequences in living mouse embryonic stem cells with a programmable fluorescent CRISPR/Cas system. Nucleus *5*, 163–172. https://doi.org/10.4161/nucl.28488.
82. Ma, H., Naseri, A., Reyes-Gutierrez, P., Wolfe, S.A., Zhang, S., and Pederson, T. (2015). Multicolor CRISPR labeling of chromosomal loci in human cells. Proc. Natl. Acad. Sci. USA *112*, 3002–3007. https://doi.org/10.1073/pnas.1420024112.
83. Xu, Q., Schlabach, M.R., Hannon, G.J., and Elledge, S.J. (2009). Design of 240,000 orthogonal 25mer DNA barcode probes. Proc. Natl. Acad. Sci. USA *106*, 2289–2294. https://doi.org/10.1073/pnas.0812506106.
84. Green, M.R., and Sambrook, J. (2019). Isolation of DNA Fragments from Polyacrylamide Gels by the Crush and Soak Method. Cold Spring Harb. Protoc. *2019*, prot100479. https://doi.org/10.1101/pdb.prot100479.
85. Heine, J., Reuss, M., Harke, B., D'Este, E., Sahl, S.J., and Hell, S.W. (2017). Adaptive-illumination STED nanoscopy. Proc. Natl. Acad. Sci. USA *114*, 9797–9802. https://doi.org/10.1073/pnas.1708304114.
86. Schindelin, J., Arganda-Carreras, I., Frise, E., Kaynig, V., Longair, M., Pietzsch, T., Preibisch, S., Rueden, C., Saalfeld, S., Schmid, B., et al. (2012). Fiji: an open-source platform for biological-image analysis. Nat. Methods *9*, 676–682. https://doi.org/10.1038/nmeth.2019.
87. Bahry, E., Breimann, L., Zouinkhi, M., Epstein, L., Kolyvanov, K., Mamrak, N., King, B., Long, X., Harrington, K.I.S., Lionnet, T., and Preibisch, S. (2022). RS-FISH: precise, interactive, fast, and scalable FISH spot detection. Nat. Methods *19*, 1563–1567. https://doi.org/10.1038/s41592-022-01669-y.

CellPress
OPEN ACCESS

STAR★METHODS

KEY RESOURCES TABLE

REAGENT or RESOURCE	SOURCE	IDENTIFIER
Chemicals, peptides, and recombinant proteins		
5-Propargylamino-dCTP-ATTO-488 (1 mM)	Jena Bioscience	Cat# NU-809-488
5-Propargylamino-dCTP-ATTO-594 (1 mM)	Jena Bioscience	Cat# NU-809-594
5-Propargylamino-dCTP-ATTO-647N (1 mM)	Jena Bioscience	Cat# NU-809-647N-S/L
5-Propargylamino-dCTP-Cy3	Jena Bioscience	Cat# NU-809-CY3-S/L
Diamond^{TM} Nucleic Acid Dye	Promega	Cat# H1181
DiYO-1	AAT Bioquest	Cat# 17580
dNTP Set (100 mM)	Thermo Fisher Scientific	Cat# R0181
Formaldehyde (16%)	Polysciences	Cat# 18814-10
Formamide ≥99.5%	Sigma Aldrich	Cat# F9037
Klenow Fragment (exo-)	Thermo Fisher Scientific	Cat# EP0421
Lambda exonuclease	Thermo Fisher Scientific	Cat# EN0561
Phusion® High-Fidelity DNA Polymerase (Phusion)	New England BioLabs	Cat# M0530 S/L
Q5® High-Fidelity DNA Polymerase (Q5)	New England BioLabs	Cat# M0491 S/L
RNase A	Thermo Fisher Scientific	Cat# EN0531
Taq DNA Polymerase (Taq)	Thermo Fisher Scientific	Cat# EP0401
Therminator^{TM} DNA Polymeras	New England BioLabs	Cat# M0261 S/L
Critical commercial assays		
Monarch® PCR & DNA Cleanup Kit	New England BioLabs	Cat# T1030 S/L
NucleoSpin Gel and PCR Clean-up Kit	Macherey-Nagel	Cat# 740609
Deposited data		
Uncropped polyacrylamide gels	This paper	https://doi.org/10.17632/nskmtr4h9y.1
Experimental models: Cell lines		
IMR-90	Coriell Biorepository	I90-79
J1	ATCC	SCRC-1010
K562	ATCC	CCL-243
U2OS	ATCC	HTB-96
Oligonucleotides		
See Table S1		
Software and algorithms		
Fiji RRID:SCR_002285	Open Source	https://fiji.sc/
Illustrator CC 2023 RRID:SCR_010279	Adobe	www.adobe.com
ImageJ2 (v.1.54h) RRID:SCR_003070	NIH	www.ImageJ.net/
Microsoft Excel	Microsoft	N/A
Pymol (v.2.5.5) RRID:SCR_000305	Schrödinger	https://pymol.org/2/
UCSF ChimeraX (v.1.17.3) RRID:SCR_015872	UCSF	www.rbvi.ucsf.edu/chimera/

RESOURCE AVAILABILITY

Lead contact

Further information and resource requests can be directed to and will be fulfilled by the lead contact Heinrich Leonhardt (h.leonhardt@lmu.de).

Materials availability

NOVA probes were generated using commercially available reagents and services. Sequences and detailed synthesis instructions for generating the probes reported in this study are listed in Table S1 and the Method Details.

Data and code availability

- The uncropped polyacrylamide gels have been deposited at Mendeley Data and are publicly available as of the date of publication. An accession number is listed in the Key Resources Table.
- This paper does not report original code.
- Any additional information required to reanalyze the data reported in this paper is available from the lead contact upon request.

METHOD DETAILS

Cell culture

K562 cells were cultivated in Dulbecco's modified Eagle's medium (DMEM), 10% fetal bovine serum (FBS), 100 U/mL penicillin and 100 μg/mL streptomycin. U2OS cells were maintained in McCoy's 5A medium supplemented with 10% FBS, 100 U/ml penicillin, and 100 μg/mL streptomycin at 37 °C in 5% CO_2. IMR-90 cells were cultured in DMEM, 20% FBS, 1× MEM Non-essential amino acids, and 100 U/mL penicillin, 100 μg/mL streptomycin.

Mouse embryonic stem cells (ESCs) were maintained on culture dishes treated with 0.2% gelatin in DMEM containing 16% FBS, 0.1 mM β-mercaptoethanol, 2 mM L-glutamine, 1× MEM Non-essential amino acids, 100 U/mL penicillin, 100 μg/mL streptomycin, homemade recombinant LIF, and 2i (1 μM PD032591 and 3 μM CHIR99021). For imaging, ESCs were seeded on plates that have been pre-treated with Geltrex diluted 1:100 in DMEM/F12 overnight at 37 °C in 5% CO_2. Cells were passaged every 2–4 days. All cell lines were regularly tested for Mycoplasma contamination by PCR.

Probe design

All generated probe sets are listed in Table S1. NOVA-probes labeling murine major satellites and human repetitive regions (chrX (p11.1) or chr13 (q34), telomeres) were adapted from previously published sequences.[81,82] Target regions ("A": chr11:55810891-55816978 "B": chr11:55817064-55821430) were chosen in hg38 and 60 unique oligonucleotides were selected and filtered, respectively.[24] Barcodes of xNOVA-probes containing repetitive sequences (10-mers) were obtained from previous published data.[28] To generate non-repetitive barcodes, pairs of orthogonal sequences from[83] were merged. Then, the barcodes were filtered for those containing cytosines every 10 bases and trimmed to the required length.

NOVA-FISH Probe synthesis

5′-phosphorylated templates and unlabeled primers were ordered from IDT or Eurofins. Equimolar amounts of 5′-phosphorylated templates and primers (0.10–0.17 nmol each) were combined to a final concentration of 1 μg DNA/μL in 1x ThermoPol Reaction Buffer.

The annealing temperatures were adjusted to the length of the primers. For NOVA-probes (40 nt long templates, 20 nt long primers), the sample was heated up to 95°C for 5 min followed by a stepwise cool-down (1°C/minute) to room temperature. For xNOVA-probes or xNOVA-pools (50–70 nt long templates, 40 nt long primers) the sample was heated up to 95°C for 5 min followed by a stepwise cool-down (1°C/2 min) to 60°C. Complex xNOVA-probe sets were synthesized by adding 2-fold excess of primer sets (e.g., primer 31–40 against "A") to the template pool.

NOVA- and xNOVA-probes were synthesized by adding 2–4 μg annealed DNA (2–4 μL of the solution) to a reaction mixture containing 0.25 mM dATP/dGTP/dTTP each, 0–0.25 mM dCTP, 0–0.25 mM dye-labeled dCTP and 3 U Therminator DNA polymerase in 1x ThermoPol Reaction Buffer (10 μL total volume). The ratios of dye-labeled dCTP to unlabeled dCTP varied depending on the desired labeling density. The reaction was carried out for 60 min at 72°C.

To remove single-stranded DNA, NucleoSpin Gel and PCR Clean-up Kit (Macherey-Nagel) was used according to the manufacturer's instructions. 9 volumes of buffer NTI (provided by the manufacturer) were added to one volume of sample before binding. After washing, the DNA was eluted twice in 22 μL ddH_2O (44 μL final volume). In the next step, 5′-phosphorylated strands were removed by adding 1 μL Lambda exonuclease (10 U/μL) and 5 μL Lambda exonuclease reaction buffer (10x) to a final volume of 50 μL and incubating for 30 min at 37°C. The synthesized probes were then purified using the Monarch PCR & DNA Cleanup Kit (New England BioLabs) according to the manufacturer's instructions and the quality was verified on denaturing 12–16% polyacrylamide gels.

Quality control and purification

The absorbance of samples was measured at 260 nm and 488 nm, 596 nm, or 647 nm depending on the incorporated fluorophore using a Nanodrop 2000 spectrophotometer (Thermo Fisher Scientific). To assess the quality of generated probes, samples were denatured in 90% formamide, 0.5% EDTA, 0.1% Xylene cyanol, 0.1% bromophenol blue and loaded onto a 12% polyacrylamide gel containing 6 M urea. The gel was incubated in 1x TBE buffer containing 1x Diamond Nucleic Acid Dye for 30 min at room temperature to visualize single-stranded DNA.

Complex probe sets labeling target region "A" or "B" were further purified following the "crush and soak" method with adaptations.[84] Briefly, segments of the polyacrylamide gel containing the band of interest were cut out and 2 volumes of a buffer containing 10 mM magnesium acetate tetrahydrate, 0.5 M ammonium acetate, 1 mM EDTA (pH 8.0) and 0.1% (w/v) SDS was added followed by incubation at 37°C for 16–24 h. The samples were then centrifuged at 13000 x g for 1 min and the supernatant was once more purified

using the Monarch PCR & DNA Cleanup Kit (New England BioLabs). We expect the "crush and soak" method to improve signal strength if low labeling densities are used during extension.

Polymerase Screens

Polymerases were tested for their ability to incorporate dCTP-ATTO488, dCTP-ATTO594, or dCTP-ATTO647N into oligonucleotides. The maximum number of incorporated dCTP-dye in the probe was eight (CATCCTGAAGGAATGGTCCATG**C**TTA**CC**TGGG **CCC**AT**CC**T).

For detailed information about the reaction conditions see Table S2. 0.1 nmol annealed DNA was added to the recommended reaction mixtures (10 μL final volume) and 5 U of the respective polymerase was added. The following temperatures were used during synthesis: Klenow exo-at 30°C, Taq at 64°C, Q5 at 64°C, Phusion at 64°C, Therminator DNA polymerase at 72°C. All reactions were carried out for 60 min and the reactions were stopped by adding 1 μL 0.5 M EDTA. We did not observe notable differences in incorporation efficiency between the reported results and reactions carried out at higher temperatures (Klenow exo-at 37°C, Taq at 72°C, Q5 at 72°C, Phusion at 72°C, Therminator DNA Polymerase at 75°C) (figure not shown). The absorbance of synthesized products was measured at 200–700 nm on Nanoquant plates using a Tecan Spark microplate reader (Tecan) and choosing the following dye-correction factors: CF_{260}(ATTO488): 0.22, CF_{260}(ATTO594): 0.22, CF_{260}(ATTO647N): 0.04. The depicted data contained at least two measurements per biological replicate.

HPLC

HPLC was used to characterize the number of incorporated fluorophores in NOVA probes with low fluorophore input in synthesis (Figures S3G and S3H). ATTO594-labeled and ATTO647N-labeled probes (0.31 nmol or 0.34 nmol) were analyzed and purified by reverse-phase HPLC using an Agilent Technologies 1260 Infinity II System with a G7165A detector equipped with an EC 250/4 Nucleodur 100-3 C18ec column from Macherey Nagel. A gradient of 0–80% of buffer B in 45 min at 60°C with a flow rate of 1 mL/min was applied. The following buffer system was used: buffer A: 100 mM NEt_3/HOAc, pH 7.0 in H_2O and buffer B: 100 mM NEt_3/HOAc, pH 7.0 in H_2O/MeCN 20/80. The fractions of each signal peak were combined, and the solvents were concentrated by vacuum centrifugation.

Sample preparation and fluorescence *in situ* hybridization

Fluorescence *in situ* hybridization was performed as previously described.[24] Adherent cells were grown overnight on glass coverslips (1.5, 18 × 18 mm, Marienfeld), washed twice with 1x Dulbecco's Phosphate Buffered Saline (PBS), and fixed using osmotically balanced and methanol-free 4% formaldehyde for 10 min at room temperature. Alternatively, PBS-washed suspension cells were resuspended in a small volume of PBS at a density of 1 million cells per mL and applied to poly-L-lysine coated glass coverslips followed by the addition of methanol-free 4% formaldehyde for 10 min at room temperature. The slides were washed twice in 1x PBS for 5 min and the cells were permeabilized in 1X PBS containing 0.5% Triton X-100 for 15 min. After two successive washing steps in 1x PBS, 0.1 M HCl was added to the slides for 5 min. The slides were washed twice with 2 x SSC and were placed onto a solution containing 1 μg/mL RNase for 30 min at 37°C in a wet chamber. Then, adherent or suspension cells were pre-equilibrated in 2x SSC containing 50% formamide for 60 min or overnight, respectively, inverted onto 8 μL of hybridization solution, and sealed with rubber cement (Marabu). The slides were placed on a heat block set to 81°C for 3 min and incubated at 37°C overnight (16–20 h).

On the second day, slides were washed twice with 2x SSC for 15 min followed by two successive 7-min washes in 0.2x SSC containing 0.2% Tween 20 at 56°C. Then, slides were washed with 4x SSC containing 0.2% Tween 20 and with 2x SSC for 5 min, respectively.

For oligoFISH probes, a second hybridization step was performed for 30 min at room temperature. The slides were then washed once with 2x SSC containing 30% formamide for 7 min at 37°C, twice with 2 x SSC for 5 min, once with 0.2X SSC containing 0.2% Tween 20 at 56°C, once with 4x SSC containing 0.2% Tween 20 for 7 min at room temperature and once with 2x SSC for 5 min.

DNA was counterstained with DAPI (1 μg/mL in 2x SSC) for 10 min and washed twice with 2x SSC. For STED microscopy, nuclei were counterstained with or DiYO-1 (12.5 nM in 2x SSC) for 30 min and washed twice with 2x SSC for 5 min, respectively. Coverslips were mounted on microscopic slides with MOWIOL (2.5% DABCO, pH 7.0), dried for 30 min, and sealed with nail polish.

Image acquisition

Confocal images were acquired using a Nikon TiE microscope equipped with a Yokogawa CSU-W1 spinning-disk confocal unit (50 μm pinhole size), an Andor Borealis illumination unit, Andor ALC600 laser beam combiner (405 nm/488 nm/561 nm/640 nm), Andor IXON 888 Ultra EMCCD camera, and a Nikon 100×/1.45 NA oil immersion objective. The microscope was controlled by software from Nikon (NIS Elements, ver. 5.02.00).

Super-resolution was carried out on a 2C STED 775 QUAD Scan microscope (Abberior Instruments) equipped with a 100x 1.4 NA UPlanSApo oil immersion objective lens (Olympus), 3 pulsed excitation lasers (485 nm, 594 nm, 640 nm) and a pulsed depletion laser of 775 nm.

3D STED microscopy of telomers using adaptive illumination

To avoid photobleaching NOVA-FISH stained telomers of IMR90 cells in 3D, stacks were acquired using adaptive illumination STED microscopy.[85] Cells were recorded using a pixel size of 30 nm, z-steps of 80 nm, a 10 μs dwell time, and a pinhole size of 50 μm.

Automated STED microscopy for two-color NOVA-FISH

Automated STED microscopy was performed according to Brandstetter et al..[24] The acquisition of 3D confocal stacks was automated using home-written Python scripts to control the microscope. Spots within confocal scans were detected using a Laplacian-of-Gaussian blob detector for both channels. Detected spots no further apart than 5 pixels from another spot in the other channel were imaged using 3D STED settings. This process was repeated for each detected spot pair within a confocal scan. Following a spiral pattern, the stage was moved to the next overview to repeat the confocal scan and the subsequent detailed STED acquisition until a specified amount of time elapsed.

Image analysis

For the analysis of the effects of labeling density (Figures 2E–2G), cells in confocal z-stacks of major satellites were segmented first via automatic thresholding in a z-maximum projection of the DAPI channel followed by a second round of thresholding in the 640nm (rel. binding) channel to segment major satellites. In the segmented areas, intensities of both the 488nm (rel. brightness) and the 640nm (rel. binding) channels were measured, background, determined by a manually selected ROI outside the cells, was subtracted, and measurements were averaged (median) per cell. For the plots, measurements were normalized to the intensity at 100% for the binding channel and at 25% for the brightness channel. Analysis was carried out using Fiji.[86]

For analysis of image data of repetitive and non-repetitive loci (in Figures 1D–1F; Figures 3B and 3C, Figures 3F and 3G; Figures 4D; S4C), nuclear segmentation maps of confocal images stained with DAPI or DiYO-1 were obtained using Otsu thresholding. FISH spots within segmentation maps were detected using a Laplacian-of-Gaussian blob detector (Figure 3F and 3G; Figure S4C). Alternatively, FISH spots were detected in each channel using RSFISH[87] and detection threshold parameters were adjusted if necessary (in Figures 1D–1F; Figures 3B and 3C; Figure 4D). Segmentation maps were used to calculate the total number of spots per cell, to obtain the mean background signal within single nuclei to calculate the spot signal over the nucleus background, and the signal-to-noise ratio of single spots. For Figure 4D, distances <500 nm between A and B were considered co-localizing.

Analysis of automated STED measurements of FISH spot pairs was performed as previously described.[24] Automated image acquisition generated large quantities of data requiring an additional quality control step. To filter out low-quality images, we used a machine learning-based classifier (Random Forest) to label images as "good" or "bad". The classifier was trained with a ground truth dataset created by an experienced scientist who manually sorted images.

Detailed spot analysis was performed on images passing this QC step. Subpixel localization of FISH spots in both channels was performed by fitting a multidimensional Gaussian function plus a constant background using the Levenberg-Marquardt algorithm. The peak height of the fitted Gaussians was used to determine spot intensity.

QUANTIFICATION AND STATISTICAL ANALYSIS

The experiments shown in this study were performed as three biologically independent experiments (n = 3) and the figures contain pooled data. No statistical methods were used to predetermine the sample size. Images depicted are representative images from the experiments and dotted lines indicate the outlines of the cells. Data plotted as boxplots indicate the 25th and 75th percentiles, with the whiskers showing the minima and maxima (5th and 95th percentiles), black circles indicating the outliers, and the horizontal line showing the median. Some data are plotted in bar graphs as the mean ± SD. Data was normalized by the median of the first depicted condition in the replicates, if not stated otherwise. Significance levels were tested by non-parametric two-sided Wilcoxon tests or pairwise comparisons using the Wilcoxon rank-sum test with Bonferroni's correction for multiple testing (* = $p < 0.05$, ** = $p < 0.01$, *** = $p < 0.001$). Sample sizes for all of the graphs are indicated in the figures or figure legends.

Cell Reports Methods, Volume *4*

Supplemental information

Generation of densely labeled oligonucleotides for the detection of small genomic elements

Clemens Steinek, Miguel Guirao-Ortiz, Gabriela Stumberger, Annika J. Tölke, David Hörl, Thomas Carell, Hartmann Harz, and Heinrich Leonhardt

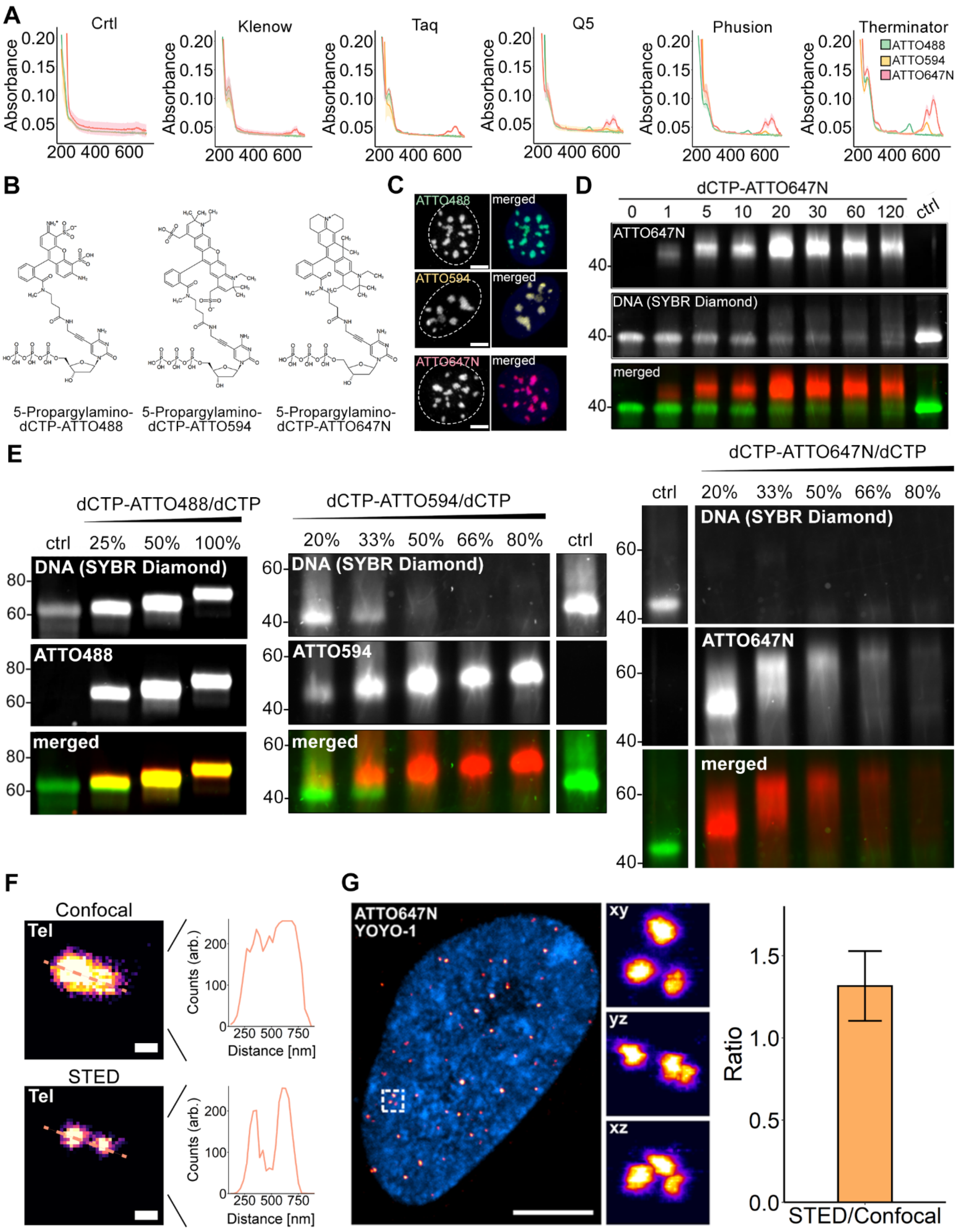
A
Crtl
Klenow
Taq
Q5
Phusion
Therminator
ATTO488
ATTO594
ATTO647N
Absorbance
0.20
0.15
0.10
0.05
200 400 600
B
5-Propargylamino-dCTP-ATTO488
5-Propargylamino-dCTP-ATTO594
5-Propargylamino-dCTP-ATTO647N
C
ATTO488
ATTO594
ATTO647N
merged
D
dCTP-ATTO647N
0 1 5 10 20 30 60 120 ctrl
ATTO647N
DNA (SYBR Diamond)
merged
40
E
dCTP-ATTO488/dCTP
ctrl 25% 50% 100%
dCTP-ATTO594/dCTP
20% 33% 50% 66% 80% ctrl
dCTP-ATTO647N/dCTP
ctrl 20% 33% 50% 66% 80%
DNA (SYBR Diamond)
ATTO488
ATTO594
ATTO647N
merged
80
60
40
F
Confocal
STED
Tel
Counts (arb.)
200
100
0
250 500 750
Distance [nm]
G
ATTO647N
YOYO-1
xy
yz
xz
Ratio
1.5
1.0
0.5
0.0
STED/Confocal

Supplementary Figure 1 Synthesizing NOVA-probes harboring different fluorophores. Related to Figure 1. (A) Absorption spectra of synthesized probes. The line depicts the mean absorption and the area indicates the standard deviation of three experiments with at least two separate measurements (n = 3). The incorporated nucleotides are indicated as follows: ATTO488 = green, ATTO594 = yellow, ATTO647N = red. (B) Structural formulas of used modified nucleotides. Dyes (ATTO488, ATTO594, ATTO647N) are linked to 5C positions of cytosines. The structures were provided by the manufacturer (Jena Bioscience). (C) Labeling major satellites in J1 cells with NOVA-FISH using three different fluorophores. All probes (maj. Sat.-ATTO488, maj. Sat.-ATTO594, maj. Sat.-ATTO647N) were generated using a one to four molar ratio of dye-labeled to unlabeled nucleotides. Scale bars, 5 µm (D) Therminator DNA polymerase effectively generates dye-labeled probes. The synthesis was carried out between 0-120 minutes using 0.15 nmol DNA and 3 U Therminator polymerase. Fluorescent DNA (ATO647N) and DNA (SYBR-Diamond) are shown in red and green, respectively. (E) Generating probes with different labeling densities. Left: Synthesis of ATTO488-labeled probes detecting major satellites. Middle: Synthesis of ATTO594-labeled probes targeting a subtelomeric region in chromosome 13. Right: Synthesis of ATTO647N-labeled probes detecting a subtelomeric region in chromosome 13. Different dye-labeled dCTP to dCTP ratios were used (25%, 50%, 100% or 20%, 33%, 50%, 66% 80%). Unlabeled probes were used as a control. Fluorophores and stained DNA (SYBR Diamond) are shown in red and green, respectively. (F) Super-resolution microscopy uncovers clustered telomeres. Representative image of two clustered telomeres using confocal microscopy or STED microscopy. Scale bars, 200 nm. (G) Telomere clustering is a common phenomenon in mitotic cells. Representative image of telomeres in IMR-90 cells using 3D-STED microscopy. Detailed view (white box) in three dimensions. Scale bar, 5 µm. Ratio of telomeres detected with STED or confocal microscopy in the same cells is depicted. Telomeres were counted in 10 individual cells from three experiments (n = 3). The black lines depict the mean.

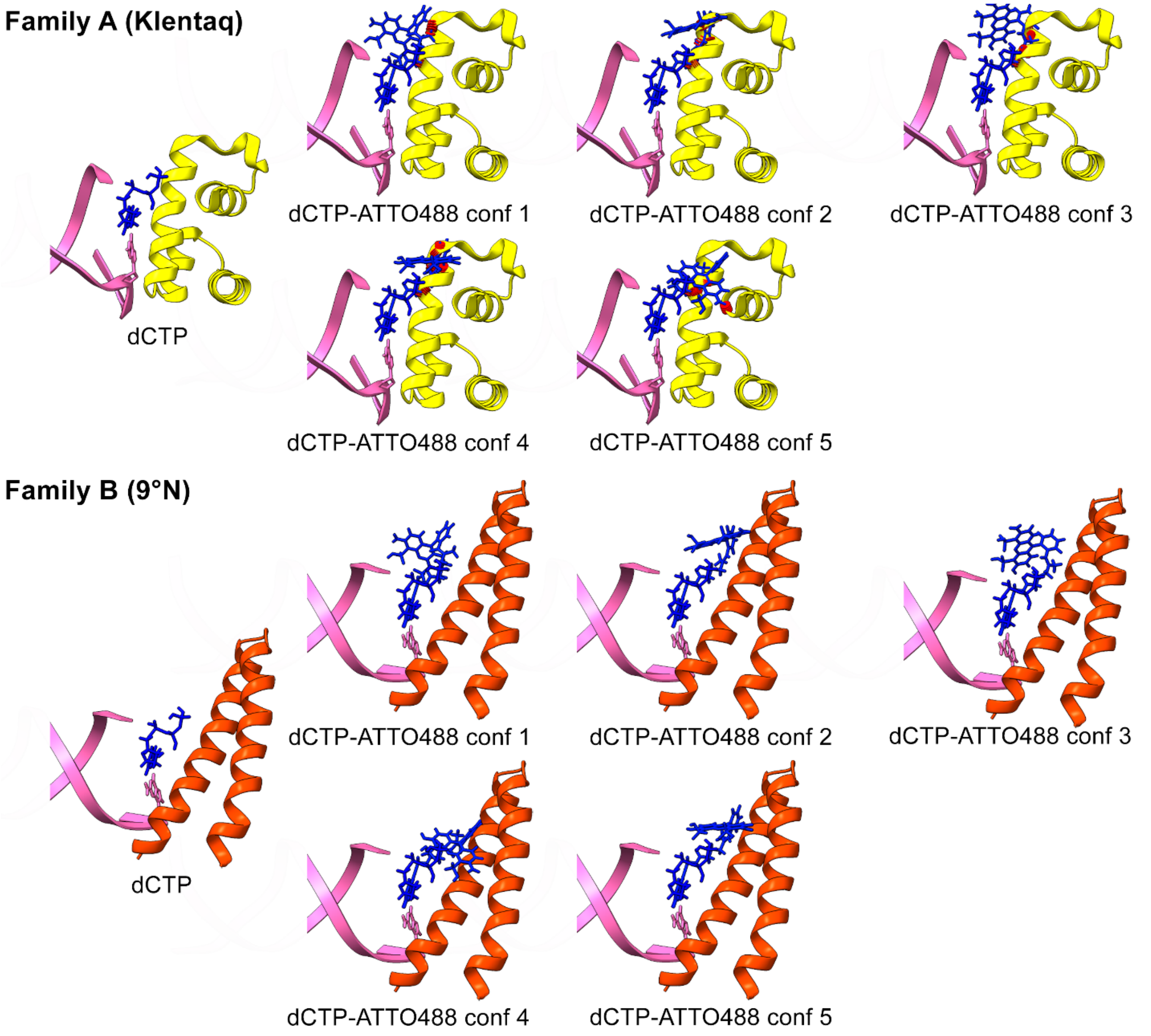

Supplementary Figure 2 Modeling the proximity of dCTP-ATTO488 to family A or family B finger domains. Related to Figure 1. Finger domains are shown in yellow (Klentaq) and orange (9°N DNA polymerase) with DNA in pink and the substrate in blue. Five different conformations of dCTP-ATTO488 (conf1-5) were superimposed on cytosine. Distances between the finger domain and dCTP-ATTO488 < 1 Å are depicted as red knobs. The finger domains are shown in the closed state. The figure was generated with UCSF Chimera (v.1.17.3, RRID:SCR_015872) by using the structures 3RTV and 5OMV [S1-S3].

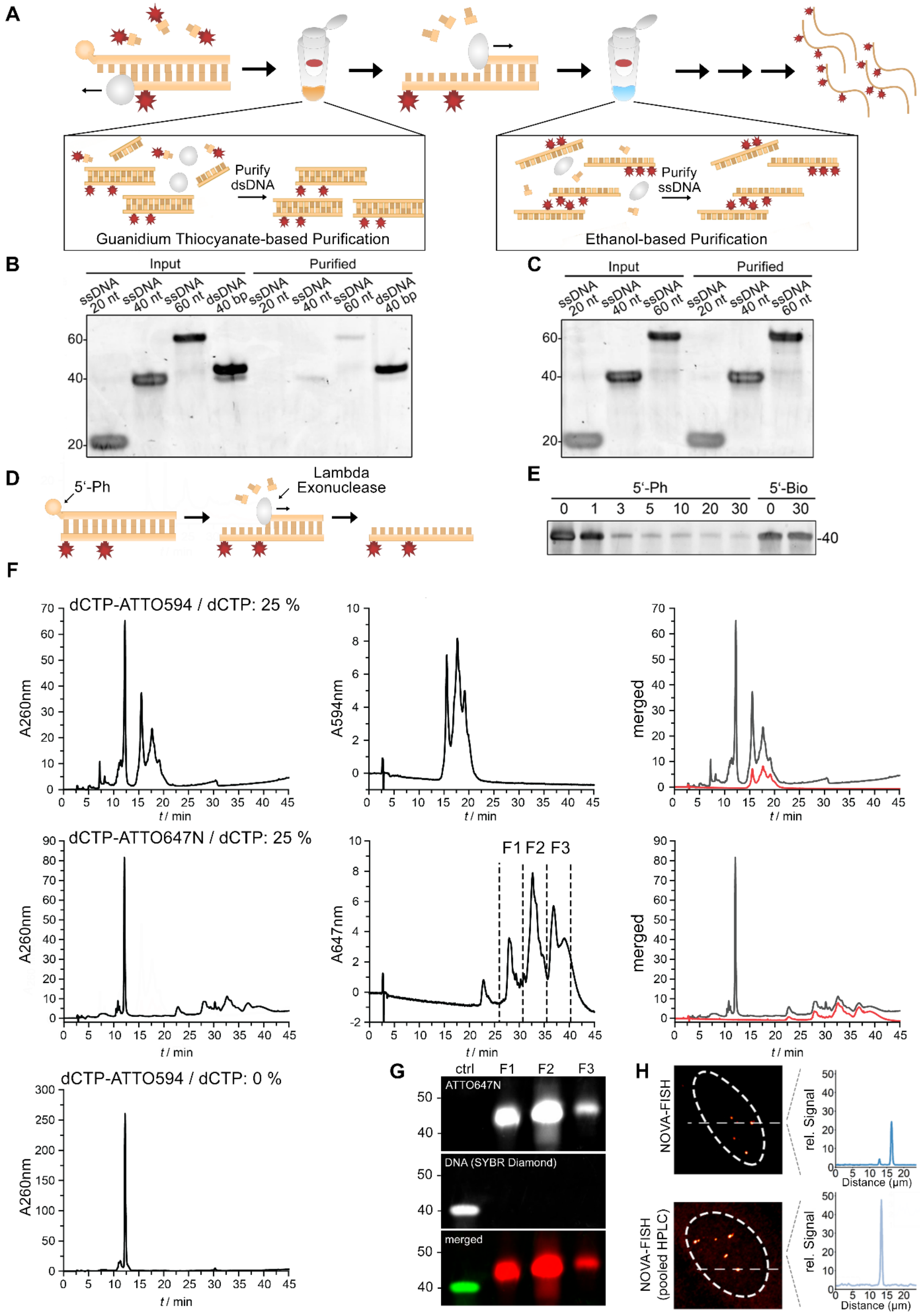
A
Purify dsDNA
Guanidium Thiocyanate-based Purification
Purify ssDNA
Ethanol-based Purification
B
Input
Purified
ssDNA 20 nt
ssDNA 40 nt
ssDNA 60 nt
dsDNA 40 bp
C
D
5'-Ph
Lambda Exonuclease
E
5'-Ph
5'-Bio
0 1 3 5 10 20 30 0 30
-40
F
dCTP-ATTO594 / dCTP: 25 %
A260nm
A594nm
merged
t / min
dCTP-ATTO647N / dCTP: 25 %
A647nm
F1 F2 F3
dCTP-ATTO594 / dCTP: 0 %
G
ctrl F1 F2 F3
ATTO647N
DNA (SYBR Diamond)
merged
H
NOVA-FISH
NOVA-FISH (pooled HPLC)
rel. Signal
Distance (µm)

Supplementary Figure 3 Optimization of probe purification. Related to STAR Methods. (A) Schematic highlighting two purification steps in the protocol. While guanidium thiocyanate-based purification enriches double-stranded DNA, ethanol-based purification purifies single-stranded oligonucleotides. (B) Guanidium thiocyanate-based purification yields double-stranded DNA. ssDNA (20 nt, 40 nt, 60 nt) and dsDNA (40 bp) were loaded before (input) and after (purified) purification. (C) Ethanol-based purification yields single-stranded DNA. ssDNA (20 nt, 40 nt, 60 nt) was loaded before (input) and after (purified) purification. (D) Schematic of lambda exonuclease-mediated degradation. Lambda exonuclease selectively removes 5'-phosphorylated (5'-Ph) strands. (E) Removal of the 5'-phosphoryated template. Templates were incubated with 10 U lambda exonuclease. 5-biotinylated oligonucleotides were used as a control. (F) HPLC of NOVA-FISH probes. Probes were synthesized with Therminator DNA Polymerase using a one to four molar ratio of dye-labeled to unlabeled nucleotides (highest possible number of incorporated fluorophores: 8). The absorptions of ATTO594-labeled and ATTO647N-labeled probes were measured at 260 nm / 594 nm and 260 nm / 647 nm, respectively. Local peaks indicate probes with varying numbers of fluorophores. Probes synthesized without modified nucleotides were used as a control (left). (G) Polyacrylamide gel reveals a visible shift between populations with different fluorophore numbers. Three fractions (F1, F2, F3) were isolated from (F) through preparative HPLC. The precise mechanism of SYBR-Diamond-ssDNA interactions has yet to be elucidated but we observed that the presence of fluorophores in the probe impacts staining efficiency. Unlabeled DNA was used as a control (ctrl). (H) Side-by-side comparison of unpurified and purified NOVA-probes. To evaluate the benefits of HPLC purification, we conducted FISH in U2OS cells using NOVA-probes before and after HPLC purification. The relative signal intensity along the dotted line is depicted. Images were acquired at the same conditions.

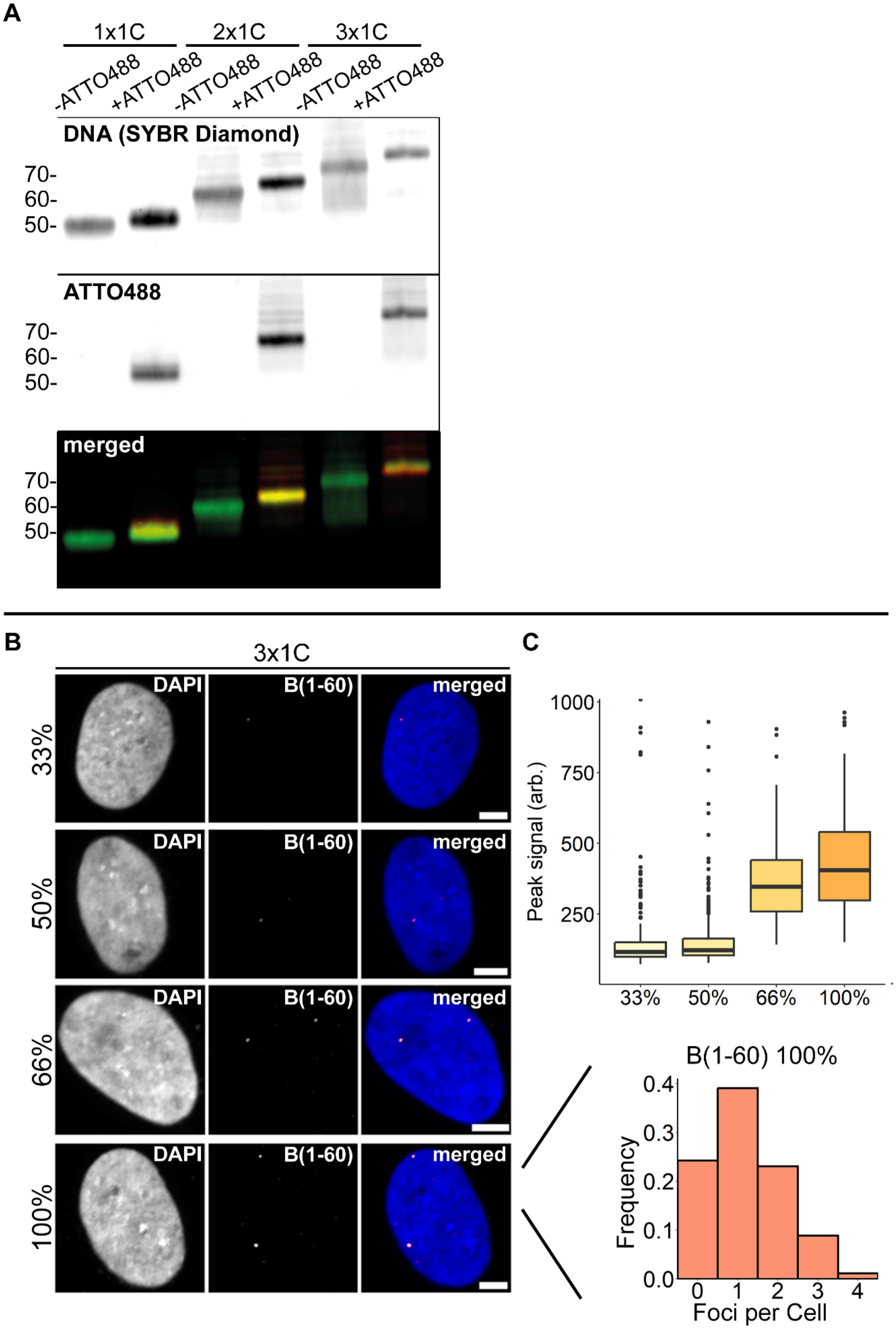
A
1x1C
2x1C
3x1C
-ATTO488
+ATTO488
-ATTO488
+ATTO488
-ATTO488
+ATTO488
DNA (SYBR Diamond)
70-
60-
50-
ATTO488
70-
60-
50-
merged
70-
60-
50-
B
3x1C
DAPI
B(1-60)
merged
33%
50%
66%
100%
C
Peak signal (arb.)
1000
750
500
250
33%
50%
66%
100%
B(1-60) 100%
Frequency
0.4
0.3
0.2
0.1
0.0
0
1
2
3
4
Foci per Cell

Supplementary Figure 4 Characterization of xNOVA-probes. Related to Figure 3. (A) Synthesis of 1C, 2x1C, or 3x1C probes carrying ATTO488 dyes. Stained DNA (SYBR Diamond) and fluorophores (ATTO488) are shown in green and red, respectively. (B) Representative confocal image of a non-repetitive region (“B”, see Figure 4A) in U2OS cells. “B” was labeled with 60 xNOVA-probes (3x1C) containing increasing labeling densities (33%, 50%, 66%, 100%). Scale bars, 5 µm. (C) xNOVA-FISH signals increase with higher labeling densities. Detected FISH intensities are displayed as boxplots. The signal was normalized by the background. Histogram of the number of foci detected in U2OS cells (n = 169). The values were normalized by the total number of cells.

	Manufacturer	Input	Costs
**NOVA-FISH**			
**1. Consumables (0.25 nmol, 100 FISH slides)**			
dNTP Mix (10 mM each)	ThermoFisher Scientific	2,5 µl	0,10 €
5-Propargylamino-dCTP-ATTO-647N (1mM)	Jena Bioscience	0,25-1,25 µl*	2,95-14,675 €*
Therminator™ DNA Polymerase	New England Biolabs	3 U	1,815 €
Monarch® PCR & DNA Cleanup Kit (250x)	New England Biolabs	1 Column	1,5 €
NucleoSpin Gel and PCR Clean-up Kit (250x)	Macherey-Nagel	1 Column	0,97 €
Thermo Scientific Lambda Exonuclease (1000 U)	ThermoFisher Scientific	10 U	1,02 €
Consumables Costs:			**8,4-20,08 €**
**2. DNA strands (for 100 Reactions)**			
**For 1 Probe:**			
Template Strand (25 nmol)	IDT	3 µg	21,66 €
Primer Strand (25 nmol)	IDT	1,5 µg	2,33 €
Total Costs:			**32.35-44.07 €**
**For 50 Probes:**			
Template Pool (50 pmol / oligo)	IDT	3 µg	106,00 €
Primer Pool (50 pmol / oligo)	IDT	1,5 µg	106,00 €
Total Costs:			**220,36-232,082 €**
**For 5 x 10 Probes:**			
Template Pool (50 pmol / oligo)	IDT	3 µg	106,00€
Primer Plate (25 nmol / oligo)	Merck	1,5 µg	220 €
Total Costs:			**367,79-426,41 €**

Supplementary Table 2 Calculated costs of NOVA-probe synthesis. Related to STAR Methods. *depending on the desired labeling density (20%, 100% fluorophore input in synthesis shown).

	Manufacturer	Yield	Costs
**oligoFISH (one-time purchase)**			
End-Labeled Secondary Probe (ATTO647N)	IDT	100 nmol***	204,97 €
**For 1 Probe:**			
Primary Probe	IDT	25 nmol	7,00 €
Total Costs:			**7,00 € (211,97 €)***
**For 50 Probes:**			
Primary Probe Pool	IDT	50 pmol/oligo	106,00 €
Total Costs:			**106,00 € (310,97 €)***
**For 5 x 10 Probes:**			
Primary Probe Plate	Merck	(25 nmol / oligo in plate)	330 €
Total Costs:			**330,00 € (534,97 €)***

Supplementary Table 3 Calculated costs of OligoFISH probes. Related to STAR Methods. *211,97 €, 310,97 €, or 534,97 € if a 3'-labeled readout probe has to be ordered.

	Manufacturer	Yield	Costs
**End-Labeled Probes (one-time purchase)**			
**For 1 Probe:**			
End-labeled Primary Probe (ATTO647N)	IDT	100 nmol*	**204,97 €**
**For 50 Probes:**			
End-labeled Primary Probe Plate (ATTO647N)	Eurofins Genomics	10 nmol / oligo	**4845 €**
**For 5 x 10 Probes:**			
End-labeled Primary Probe Plate (ATTO647N)	Eurofins Genomics	10 nmol / oligo	**4845 €**

Supplementary Table 4 Calculated costs of end-labeled probes. Related to STAR Methods.
*minimum synthesis scale

REFERENCES

S1. Pettersen, E.F., Goddard, T.D., Huang, C.C., Couch, G.S., Greenblatt, D.M., Meng, E.C., and Ferrin, T.E. (2004). UCSF Chimera--a visualization system for exploratory research and analysis. J Comput Chem *25*, 1605-1612. https://doi.org/10.1002/jcc.20084.

S2. Betz, K., Malyshev, D.A., Lavergne, T., Welte, W., Diederichs, K., Dwyer, T.J., Ordoukhanian, P., Romesberg, F.E., and Marx, A. (2012). KlenTaq polymerase replicates unnatural base pairs by inducing a Watson-Crick geometry. Nat Chem Biol *8*, 612-614. https://doi.org/10.1038/nchembio.966.

S3. Kropp, H.M., Betz, K., Wirth, J., Diederichs, K., and Marx, A. (2017). Crystal structures of ternary complexes of archaeal B-family DNA polymerases. PLoS One *12*, e0188005. https://doi.org/10.1371/journal.pone.0188005.
